# Mechanistic Pathways Controlling Cadmium Bioavailability and Ecotoxicity in Agricultural Systems: A Global Meta-Analysis of Lime Amendment Strategies

**DOI:** 10.3390/biology15030207

**Published:** 2026-01-23

**Authors:** Jianxun Qin, Keke Sun, Yongfeng Sun, Shunting He, Yanwen Zhao, Junyuan Qi, Yimin Lan, Beilei Wei, Ziting Wang

**Affiliations:** 1 School of the Earth Sciences and Resources, China University of Geosciences, Beijing 100083, China; qinjianxun@gxdzdcy.cn; 2 Guangxi Institute of Geological Survey, Nanning 530023, China; 3 Guangxi Engineering Research Center for Medical Geology, Nanning 530023, China; 4 State Key Laboratory for Conservation and Utilization of Subtropical Agri-Biological Resources, Guangxi Key Laboratory for Sugarcane Biology, College of Agriculture, Guangxi University, Nanning 530004, China; sun1967230965@163.com (K.S.); 18778977421@163.com (Y.S.); shuntinghe0319@163.com (S.H.); 15678585108@163.com (Y.Z.); 2517300022@st.gxu.edu.cn (Y.L.)

**Keywords:** cadmium bioavailability, ecotoxicological pathways, lime amendments, ionic competition, food chain transfer

## Abstract

Cadmium (Cd) contamination in agricultural systems poses significant ecotoxicological risks through bioaccumulation in food chains. While lime-based amendments are widely applied for Cd immobilization, mechanistic understanding of bioavailability control pathways remains limited. This study employed a meta-analysis methodology based on 260 datasets from 55 publications to systematically investigate the mechanisms and differences in the effectiveness of calcium hydroxide, calcium carbonate, and calcium oxide in regulating Cd migration in acidic soil–plant systems. The study revealed that lime-based materials synergistically regulated Cd migration through two processes: chemical fixation and ionic competition. Results showed lime application reduced soil available Cd by 33.0%, decreased grain Cd by 44.8%, increased soil pH by 15.6%, and enhanced exchangeable Ca by 35.2%. Chemical fixation was evidenced by Cd transformation from labile to stable forms (residual Cd: +29.5%, acid-soluble Cd: −17.5%). Ionic competition was quantitatively confirmed through strong negative correlation between exchangeable Ca and grain Cd (R^2^ = 0.704). Among the materials, Ca(OH)_2_ exhibits the highest efficiency in rapid pedogenic passivation (58.7% reduction in available Cd), whereas CaCO_3_ demonstrates superior long-term grain Cd attenuation (65.7% inhibition) via sustained Ca^2+^ release and rhizosphere-regulated dissolution. This study advances mechanistic understanding of Cd bioavailability control and establishes quantitative frameworks for predicting ecotoxicological outcomes, providing scientific basis for optimizing remediation strategies to minimize Cd transfer through agricultural food chains.

## Simple Summary

Cadmium contamination in agricultural soils threatens food safety through crop bioaccumulation, particularly in rice. Lime-based materials (calcium carbonate, calcium hydroxide, and calcium oxide) are commonly applied to mitigate cadmium uptake, yet comparative effectiveness and mechanistic understanding remain limited. This meta-analysis synthesized data from 55 studies encompassing 260 field experiments to evaluate how these three materials regulate cadmium transfer from soil to grain. Results indicate all materials reduced grain cadmium by approximately 45%, operating through dual mechanisms: chemical fixation transforms cadmium into stable soil forms, while ionic competition involves calcium blocking cadmium entry at root membranes. Calcium hydroxide demonstrated rapid soil immobilization capacity, reducing bioavailable cadmium by 59%. Calcium carbonate exhibited superior long-term grain protection (66% reduction) via sustained calcium release synchronized with critical grain-filling stages. Economic analysis revealed calcium carbonate provides 5–10 fold better cost-effectiveness than alternatives. These findings enable targeted material selection: calcium hydroxide for emergency soil remediation versus calcium carbonate for sustained crop protection. This mechanistic framework advances cadmium remediation from empirical application toward process-based strategies that integrate geochemical stabilization with plant physiological protection.

## 1. Introduction

Soil cadmium (Cd) contamination has emerged as a major environmental issue threatening global agricultural sustainability and food security [1]. Recent global assessments reveal that 14–17% of agricultural lands are affected by heavy metal pollution, with approximately 0.9–1.4 billion people residing in high-health-risk areas [2]. Globally, Cd is the most widely distributed soil contaminant, with approximately 9% of agricultural soils exceeding safe Cd thresholds (e.g., 0.3–0.6 mg/kg for agricultural soils depending on pH, according to various international guidelines) [3]. As a Group I carcinogen confirmed by the World Health Organization, Cd possesses the unique characteristic of accumulating in edible plant tissues without significantly affecting plant growth, enabling “cryptic” transfer into the food chain and causing long-term chronic health hazards [4,5]. Rice, which serves as the staple food for more than 3 billion people worldwide, exhibits particularly prominent grain Cd contamination issues, constituting the primary source of Cd exposure in human populations [6,7]. Therefore, there is an urgent need to develop multi-mechanism synergistic remediation strategies that simultaneously achieve Cd immobilization in paddy fields and inhibit Cd uptake by plants.

Calcium (Ca) plays crucial roles in various physiological and biochemical processes in plants, including cell wall composition, membrane permeability, and signal transduction [8]. In addition, Ca participates in multiple stress responses, such as drought, salinity, cold, and heavy metal stress [9]. Numerous studies have demonstrated that Ca addition can reduce Cd uptake and enhance plant tolerance to Cd, which are attributed to the multiple interactions between Ca and Cd [10]. Application of Ca-containing minerals can form Cd–Ca complexes, thereby reducing Cd availability in soil and subsequently decreasing Cd bioaccumulation in plants [11]. Due to the similar ionic radii and charges of Cd ions (Cd^2+^) and Ca ions (Ca^2+^), they can compete for the same channels/transporters at root level [12]. Therefore, applying highly available Ca helps alleviate Cd toxicity by reducing Cd uptake in crops [13]. However, the mechanisms by which lime-based materials affect Cd transport and distribution within crops remain unclear, particularly with regard to the way in which Ca ions regulate Cd uptake in crop roots and the distribution processes among different organs, for which in-depth investigations are lacking [14,15]. Furthermore, differences in effectiveness among various lime-based materials in regulating crop Cd accumulation and their underlying mechanisms require systematic elucidation, as they directly impact the effectiveness and reliability of lime-based passivation technology under different crop and soil conditions.

Existing research exhibits significant limitations in systematic evaluation of Ca-based remediation technologies. Kong et al. [16] conducted a meta-analysis on lime material remediation of Cd-contaminated paddy fields, encompassing 39 publications, and found that lime application could reduce the rice grain Cd content by 44%, and identified key influencing factors such as lime type (calcium carbonate (CaCO_3_), calcium hydroxide (Ca(OH)_2_), or calcium oxide (CaO)), dosage, and soil environmental factors. A meta-analysis by He et al. [17] confirmed that lime-based materials can significantly increase soil pH and reduce crop Cd accumulation, although these effects exhibit significant heterogeneity. Chemically, although CaCO_3_, Ca(OH)_2_, and CaO are all lime-based amendments, they differ fundamentally in their solubility, reaction stoichiometry, and impact on soil solution alkalinity [18]. CaO and Ca(OH)_2_ are strong bases that dissolve rapidly and provide a sharp increase in pH, whereas CaCO_3_ exhibits lower solubility and acts as a slow-release source of both alkalinity and Ca^2+^ ions [19]. These distinct chemical behaviors inevitably lead to varied remediation efficiencies and mechanisms in different soil environments. However, these studies still present significant limitations. Most focus solely on an overall evaluation of lime effects, lacking systematic comparison of effectiveness differences among different lime-based materials such as CaCO_3_, Ca(OH)_2_, and CaO (Table 1). Furthermore, mechanism analysis remains confined to the single perspective of pH regulation, failing to thoroughly elucidate the synergistic effects of dual mechanisms involving chemical fixation and ionic competition. In addition, the absence of quantitative relationship analysis between soil exchangeable Ca ions and plant Cd accumulation has resulted in lime-based passivation technology remaining at the empirical application stage. Meta-analysis is an important evidence-based research method that extracts universally applicable patterns by integrating numerous independent research results [20]. A systematic comparison of differences in effectiveness among different lime-based materials in regulating Cd migration through large-scale data integration provides a foundation for scientific selection of lime-based materials, which holds significant practical importance.

This study involved collecting data from 55 relevant studies based on a systematic literature review and employing a meta-analysis methodology to systematically compare the Cd inhibition effects of lime-based passivation technology. The research objectives were to (1) systematically compare the Cd inhibition effects of three major lime-based materials (CaCO_3_, Ca(OH)_2_, and CaO) and validate the synergistic effects of dual mechanisms involving chemical fixation and ionic competition; (2) analyze the quantitative relationship between soil exchangeable Ca ions and plant Cd accumulation to elucidate the functional patterns of ionic competition mechanisms; and (3) resolve dose–response relationships of different lime-based materials to identify relatively effective dosage ranges and key regulatory factors. This study aimed to provide a scientific foundation for material selection in agricultural Cd pollution remediation through systematic comparison of the effectiveness of different lime-based materials.

## 2. Materials and Methods

### 2.1. Data Sources and Treatments

Scientific publications spanning 2000–2023 were retrieved from Springer Link and Science Direct databases. Search strategies employed various keyword combinations, including “lime AND Cd AND soil,” “limestone AND Cd AND soil,” “quicklime AND Cd AND soil,” and “hydroxide calcium AND Cd AND soil”.

The selected studies focused on lime-based compounds (CaCO_3_, Ca(OH)_2_, and CaO) as immobilization agents for Cd-contaminated soil remediation and crop Cd reduction. This analysis exclusively incorporated data from field-scale and pot experiments, and omitted laboratory-scale investigations such as incubation or leaching tests. This exclusion was implemented to ensure that the integrated results reflect field-realistic conditions and capture the complex soil–plant interactions that are often absent in simplified laboratory settings.

To minimize selection bias, four specific inclusion criteria were established:

(1) Monitoring requirements: Studies must document soil pH levels, bioavailable Cd concentrations, and Cd accumulation in plant tissues (shoots and grains) of at least one crop species.
(2) Research focus: Investigations should emphasize effects of Cd contamination on agricultural crops, with particular attention to rice (*Oryza sativa* L.).
(3) Treatment design: Research must clearly specify the lime-based amendment dosages, utilize these materials as primary immobilization agents, and include both untreated controls and various Ca-amended treatments.
(4) Data availability: Essential statistical information, including sample numbers, mean values, standard deviations, and/or standard errors (SEs) for target parameters, must be explicitly presented in manuscripts, tables, or extractables from published figures.

When the *SE* was reported, the standard deviation (*SD*) was calculated using Equation (1) [22].

(1) $$SD=SE\times \sqrt{n}$$

The final database included 55 studies and 260 datasets that satisfied the above criteria (Text S1).

We calculated the theoretical value for Ca ions for lime-based material application using Equation (2) [17]:

(2) $${Ca}^{2+}\;theoretical\;value=\frac{M_{{Ca}^{2+}}}{M_{Raw\;material}}\times Application\;rate$$

where *M*_Ca_^2+^ represents the relative atomic mass of Ca ions (g/mol), *M_Raw material_* represents the molar mass of the main component in the lime-based material (g/mol), and *Application rate* represents the application rate of the lime-based material (kg/ha).

### 2.2. Meta-Analysis

Effects of nitrogen application on each variable were calculated using the natural logarithm of the response ratio [ln(*RR*)] (Equation (3)):

(3) $$\ln\left(RR\right)=\frac{X_{t}}{X_{c}},$$

where *X_t_* and *X_c_* are the mean values of the variables in the nitrogen treatment and control groups, respectively.

The mixed-effect models in the “*metafor*” package in R software (version 4.3.0) was used to calculate the weighted effect size [ln(*RR*_++_)] and 95% confidence interval (Cl) [23]:

(4) $$ln\left({RR}_{++}\right)=\frac{\sum_{i=1}^{m}\sum_{j=1}^{k}\left[ln\left(RR\right)\right]}{\sum_{i=1}^{m}\sum_{j=1}^{k}w_{ij}}$$

(5) $$S_{ln\left({RR}_{++}\right)}=\frac{1}{\sqrt{\sum_{i=1}^{m}\sum_{j=1}^{k}w_{ij}}},\;\mathrm{and},\;\mathrm{and}$$

(6) $$95\%CI=ln\left({RR}_{++}\right)+1.96\times S_{ln\left({RR}_{++}\right)}$$

where the weight of each ln(*RR*) (*w*) is the reciprocal of its variance; $S_{ln\left({RR}_{++}\right)}$ is the SE of ln(*RR*_++_); 95% CI is the 95% CI of ln(*RR*_++_); *m* is the number of groups; *i* and *j* represent the *i*-th and *j*-th treatments, respectively, and; *k* is the number of observations in the corresponding group.

The effect of nitrogen application was considered significant if the 95% CI did not overlap with zero [24]. For ease of interpretation, ln(*RR*_++_) and its 95% CI were transformed into the percentage change using Equation (7).

(7) $$Percentage\;change\;\left(\%\right)=\left[e^{ln\left({RR}_{++}\right)}-1\right]\times 100$$

### 2.3. Statistical Analysis

The R package “ggplot2” (R v4.2.3, http://www.r-project.org) was used to perform linear regression analysis. The significance of moderators was tested using the Q statistical test (Qm), with a Qm *p*-value < 0.05 indicating a significant influence of the factors on the variable response to the indicators. Rosenberg’s method was used to assess publication bias (Table S1), and pairwise correlations between ln(*RR*) values were calculated. If Rosenberg’s fail-safe number exceeded 5*n* + 10 (where *n* is the sample size), there was no publication bias in the variables [25].

## 3. Results

### 3.1. Lime-Based Materials Regulate Cd Migration in Acidic Soil–Plant Systems Through Chemical Fixation and Ionic Competition

**Figure 1:**
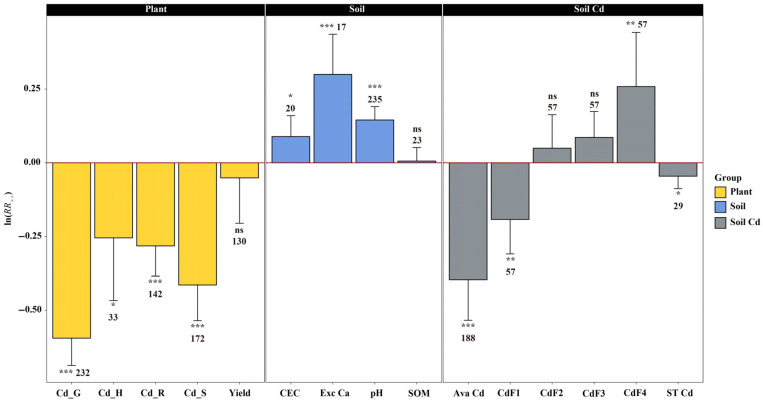
The responses of variables to lime-based materials in soil and plant. Vertical error bars indicate 95% confidence intervals calculated from the standard errors of the pooled effect sizes. The Arabic numerals at the top side represent the sample size of the response variables. The *x*-axis displays ln(RR_++_); percentage changes reported in the text were calculated using Equation (7): Percentage change (%) = [e^ln(RR_++_)^ − 1] × 100. Cd_G, cadmium content in grain; Cd_H, cadmium content in husk; Cd_R, cadmium content in root; Cd_S, cadmium content in stem; CEC, soil cation exchange capacity; Exc Ca, soil exchangeable calcium content; SOM, soil organic matter; Ava Cd, available cadmium; CdF1, Acid-soluble fraction of cadmium; CdF2, reducible fraction of cadmium; CdF3, oxidizable fraction of cadmium; CdF4, residual fraction of cadmium; ST Cd, total cadmium in soil. Significant levels at * *p* < 0.05, ** *p* < 0.01, *** *p* < 0.001 and ns *p* > 0.05.

The meta-analysis results demonstrated that lime-based material application produced synergistic Cd regulation effects through two processes: chemical fixation and ionic competition in acidic soil–plant systems (Figure 1). Chemical fixation was primarily involved in transformation of soil Cd speciation. Lime-based material application significantly reduced soil Cd bioavailability, with available Cd decreasing by 33.0% (*p* < 0.001, *n* = 189) and Acid-soluble fraction of Cd (CdF1) decreasing by 17.5% (*p* < 0.01, *n* = 57); whereas, residual fraction of Cd (CdF4) significantly increased by 29.5% (*p* < 0.01, *n* = 57), indicating Cd transformation from labile to stable forms. Ionic competition is primarily reflected in inhibition of Cd uptake by plants. Cd accumulation in all plant tissues was significantly reduced, with grain Cd decreasing by 44.8% (*p* < 0.001, *n* = 232), shoot Cd decreasing by 33.9% (*p* < 0.001, *n* = 172), root Cd decreasing by 24.6% (*p* < 0.001, *n* = 141), and husk Cd decreasing by 22.5% (*p* < 0.05, *n* = 33). Simultaneously, lime-based material application significantly improved soil physicochemical properties, increasing the pH by 15.6% (*p* < 0.001, *n* = 235), enhancing exchangeable Ca by 35.2% (*p* < 0.001, *n* = 17), increasing the cation exchange capacity (CEC) by 9.4% (*p* < 0.05, *n* = 20), and increasing the grain Ca content by 9.2% (*p* < 0.001, *n* = 14).

The regression fitting analysis confirmed the synergistic effects of chemical fixation and ionic competition (Figures S1 and S2). Under the influence of lime-based materials, exchangeable Ca exhibited a significant negative correlation with available Cd (y = −0.7x + 0.168, R^2^ = 0.856, *p* < 0.001). The ionic competition process was quantitatively validated through the linear negative correlation between exchangeable Ca and grain Cd (y = −3.04x − 0.082, R^2^ = 0.704, *p* < 0.001). With increasing exchangeable Ca response values, soil pH (y = 0.605x^2^ + 0.11x + 0.0402, R^2^ = 0.885, *p* < 0.001) and grain Ca (y = 0.0736ln[x] + 0.181, R^2^ = 0.736, *p* < 0.001) significantly increased. Cd translocation within plants also exhibited distinct patterns, with grain Cd (y = 0.932x − 0.281, R^2^ = 0.534, *p* < 0.001) and shoot Cd (y = 0.946x − 0.0974, R^2^ = 0.53, *p* < 0.001) showing significant positive correlations with root Cd, indicating that root Cd uptake was the critical determinant of aboveground Cd accumulation.

### 3.2. Differential Roles of Three Lime-Based Materials in Chemical Fixation and Ionic Competition Mechanisms

**Figure 2:**
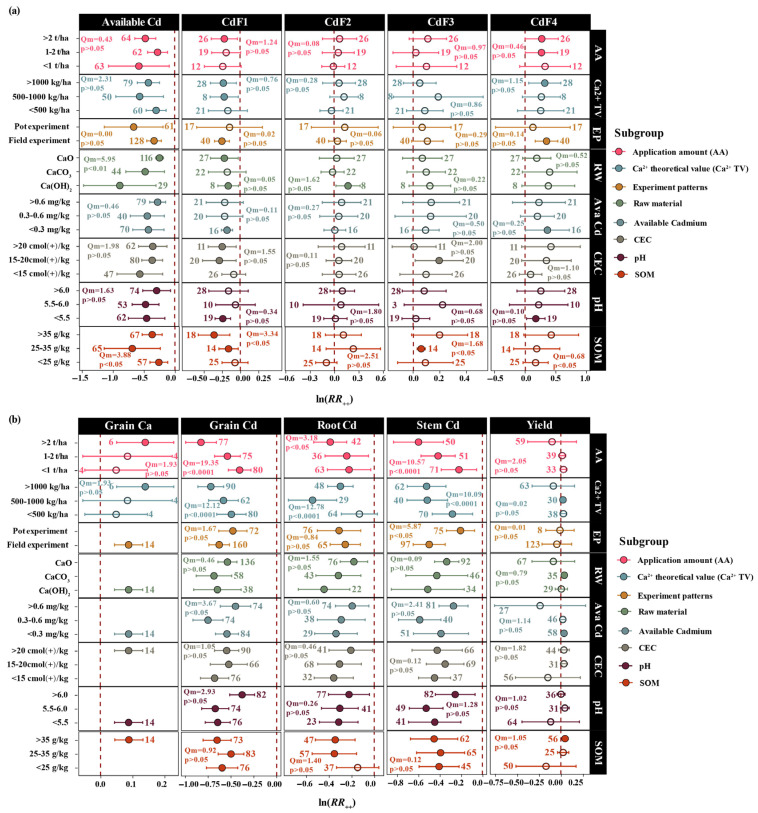
The weighted effect size of lime-based materials (**a**) in soil and (**b**) plant under different subgroups. Vertical error bars indicate 95% confidence intervals calculated from the standard errors of the pooled effect sizes. The Arabic numerals at the top side represent the sample size of the response variables. The *x*-axis displays ln(RR_++_); percentage changes reported in the text were calculated using Equation (7): Percentage change (%) = [e^ln(RR_++_)^ − 1] × 100. CdF1, Acid-soluble fraction of cadmium; CdF2, reducible fraction of cadmium; CdF3, oxidizable fraction of cadmium; CdF4, residual fraction of cadmium.

Significant differences existed in the weighting of roles among different lime-based materials within the dual mechanisms. The subgroup analysis revealed that the three lime-based materials exhibited distinct mechanistic preferences for Cd regulation (Figure 2). Ca(OH)_2_ primarily functioned through chemical fixation mechanisms and resulted in the greatest soil pH elevation (27.4%, *p* < 0.001, *n* = 33), the most significant reduction in available Cd (−58.7%, *p* < 0.01, *n* = 29), a substantial increase in exchangeable Ca (43.3%, *p* < 0.001, *n* = 14), and the strongest inhibition of root Cd (−35.7%, *p* < 0.001, *n* = 22). CaCO_3_ primarily operated through ionic competition mechanisms, exhibiting an outstanding performance in blocking plant Cd translocation, with grain Cd inhibition reaching 49.6% (*p* < 0.001; *n* = 58). CaO displayed balanced characteristics between the two mechanisms, with intermediate effects between those of the other two materials. Statistical tests revealed significant differences in the effects of the lime-based material type on soil available Cd (Qm = 5.949, *p* < 0.01), CEC (Qm = 11.889, *p* < 0.01), and soil organic matter (Qm = 260.206, *p* < 0.0001).

**Figure 3:**
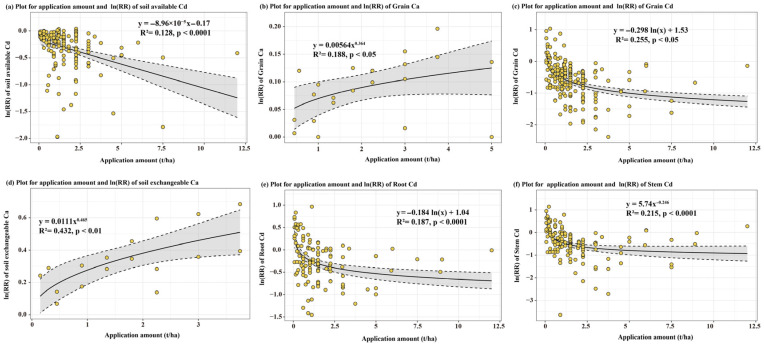
Relationships between the application amount of lime-based materials and ln(*RR*) of soil available cadmium, soil exchangeable calcium, Grain Ca, Grain Cd, Root Cd, Stem Cd. Yellow circles represent individual observed data points; the solid line denotes the fitted linear regression line; the grey shaded area represents the 95% confidence interval of the regression fit, illustrating the uncertainty associated with the predicted relationship.

Dose–response analysis revealed the staged characteristics of the dual mechanisms (Figure 2 and Figure S3). When the theoretical value for Ca^2+^ ranged from 500–1000 kg/ha, the response values of soil available Cd (−43.1%, *p* < 0.01, *n* = 50), exchangeable Ca (44.7%, *p* < 0.01, *n* = 4), and root Cd (−41.9%, *p* < 0.001, *n* = 29) to lime-based material addition exceeded those of other groups, indicating that ionic competition mechanisms were relatively active at this stage. When the theoretical value for Ca^2+^ exceeded 1000 kg/ha, the responses of CdF1 (−20.5%, *p* < 0.05, *n* = 28), CdF4 (37.9%, *p* < 0.05, *n* = 28), grain Cd (−51.4%, *p* < 0.001, *n* = 90), and grain Ca (15.1%, *p* < 0.01, *n* = 6) were more pronounced, indicating that chemical fixation mechanisms began to play a dominant role at this stage. Regression analysis demonstrated that with increasing Ca application amount (Figure 3 and Figure S4), soil available Cd (y = −8.96 × 10^−5^x − 0.17, R^2^ = 0.128, *p* < 0.0001) and grain Cd (y = −0.298ln(x) + 1.53, R^2^ = 0.255, *p* < 0.0001) exhibited significant decreasing trends, whereas exchangeable Ca (y = 0.011x^0.465^, R^2^ = 0.432, *p* < 0.01) and grain Ca (y = 0.00564x^0.364^, R^2^ = 0.188, *p* < 0.01) showed significant increasing trends. Similarly, with an increase in the theoretical value for Ca^2+^, response values of soil available Cd (y = −0.0869ln(x) + 0.255, R^2^ = 0.051, *p* < 0.01) and grain Cd (y = −0.213ln(x) + 0.796, R^2^ = 0.145, *p* < 0.0001) both exhibited significant decreasing trends, whereas response values of soil exchangeable Ca (y = 0.0451e^0.00705x^, R^2^ = 0.486, *p* < 0.01) and grain Ca (y = 0.000162x + 0.181, R^2^ = 0.379, *p* < 0.01) demonstrated significant increasing trends.

**Figure 4:**
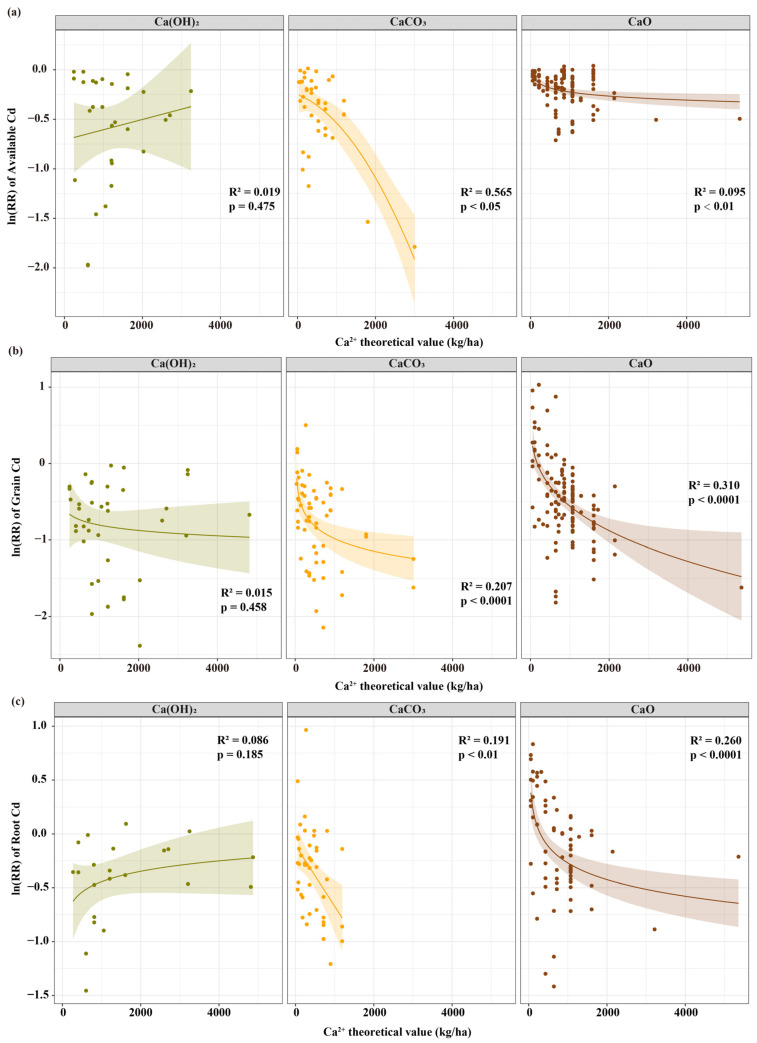
Relationships between the theoretical value for Ca^2+^ and (**a**) ln(*RR*) of soil available cadmium, (**b**) cadmium content in grain and (**c**) soil pH under the treatment of calcium hydroxide, calcium carbonate, and calcium oxide. Colored circles represent individual observed data points, with green, orange, and brown corresponding to Ca(OH)_2_, CaCO_3_, and CaO treatments, respectively. The solid line denotes the fitted regression curve for each treatment. The colored shaded area represents the 95% confidence interval of the regression fit, reflecting the uncertainty of the predicted relationship. R^2^ and *p* values in each subplot indicate the explained variance of the regression model and its statistical significance, respectively.

Mechanistic differences between the different lime-based materials were significant (Figure 4). CaO exhibited typical dose-dependent characteristics, primarily manifesting as a positive correlation between the grain Cd inhibition effects and the theoretical value for Ca^2+^ (R^2^ = 0.310, *p* < 0.001). Effects of CaCO_3_ were primarily achieved through ionic competition, with strong inhibition of soil available Cd at high doses (R^2^ = 0.565, *p* < 0.05), although the dose dependency of grain Cd was relatively weak (R^2^ = 0.207, *p* < 0.001). Ca(OH)_2_ primarily functioned through chemical fixation, with stable effects unaffected by dose variation (grain Cd: R^2^ = 0.015, *p* = 0.458), which is consistent with its chemical characteristics of rapid dissolution and immediate reactions.

### 3.3. Validation of Dual Mechanisms and Mechanistic Division of Three Lime-Based Materials

**Figure 5:**
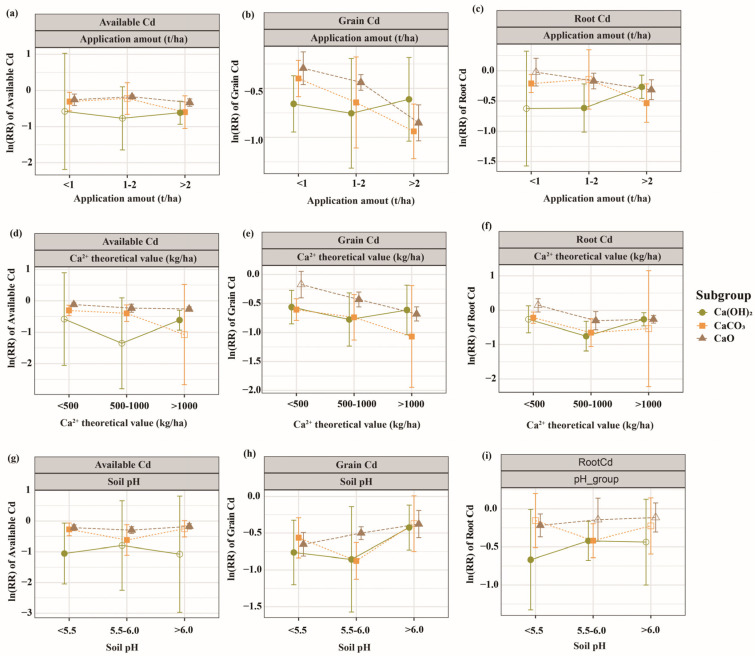
(**a**) ln(RR_++_) of soil available cadmium in relation to lime-based material application amount (t/ha). (**b**) ln(RR_++_) of grain cadmium in relation to lime-based material application amount (t/ha). (**c**) ln(RR_++_) of root cadmium in relation to lime-based material application amount (t/ha). (**d**) ln(RR_++_) of soil available cadmium in relation to theoretical Ca^2+^ application rate (kg/ha). (**e**) ln(RR_++_) of grain cadmium in relation to theoretical Ca^2+^ application rate (kg/ha). (**f**) ln(RR_++_) of root cadmium in relation to theoretical Ca^2+^ application rate (kg/ha). (**g**) ln(RR_++_) of soil available cadmium in relation to soil pH. (**h**) ln(RR_++_) of grain cadmium in relation to soil pH. (**i**) ln(RR_++_) of root cadmium in relation to soil pH. Vertical error bars indicate 95% confidence intervals calculated from the standard errors of the pooled effect sizes. The *x*-axis displays ln(RR_++_); percentage changes reported in the text were calculated using Equation (7): Percentage change (%) = [e^ln(RR_++_)^ − 1] × 100.

After categorizing Ca sources into three types, a subgroup analysis was conducted for each indicator, and further mechanistic validation analysis confirmed the collaborative division of chemical fixation and ionic competition (Figure 5). Under conditions of a high theoretical value for Ca^2+^ (>1000 kg/ha), all three lime-based materials demonstrated significant Cd inhibition, although their mechanistic contributions differed. CaCO_3_ achieved a grain Cd inhibition rate of 65.7% (*p* < 0.05, *n* = 7), primarily through ionic competition mechanisms; CaO reached 49.4% (*p* < 0.001, *n* = 64), achieved through dose-dependent dual mechanisms; and Ca(OH)_2_ attained 45.8% (*p* < 0.01, *n* = 19), primarily through chemical fixation mechanisms. The mechanistic characteristics of each lime-based material remained stable at different application rates. When application amount were < 1 t/ha, CaCO_3_ (26.5%, *p* < 0.05, *n* = 14) and CaO (22.7%, *p* < 0.01, *n* = 42) significantly reduced the available Cd content; whereas, Ca(OH)_2_ showed no significant effect on available Cd at low application rates (*p* > 0.05). When application amount exceeded 2 t/ha, the dominant mechanisms of each lime-based material were fully activated, with the ionic competition advantage of CaCO_3_ becoming more pronounced (grain Cd inhibition rate: 60.9%, *p* < 0.001, *n* = 25).

**Figure 6:**
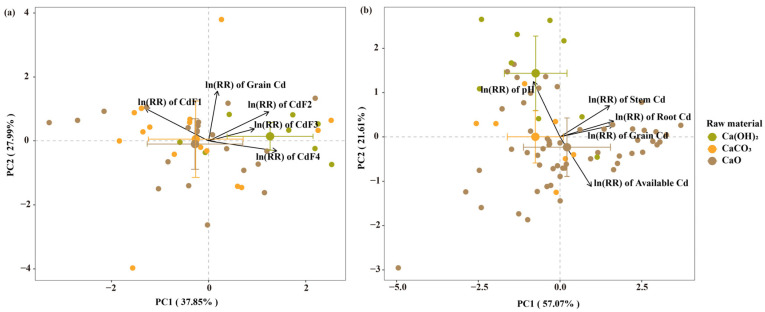
(**a**) PCA ordination of experimental samples and response variables (ln(RR) of calcium and cadmium concentrations in crop grains, and soil available cadmium fractions), showing the distribution of lime material treatments along the first two principal components (PC1 and PC2). (**b**) PCA ordination of experimental samples and response variables (ln(RR) of cadmium concentrations in crop stems and roots, and soil available cadmium), displaying the separation of lime material treatments based on PC1 and PC2.

Principal component analysis (PCA) analysis systematically revealed the mechanistic division patterns of the three lime-based materials (Figure 6a). In the PCA analysis of the soil Cd speciation distribution, the first principal component (PC1) and second principal component (PC2) explained 37.85% and 27.99% of the total variation, respectively, cumulatively accounting for 65.84% of the variation. The CaO treatment group was primarily distributed in the positive region of PC1, showing positive correlations with CdF1 and reducible fraction of Cd (CdF2), indicating that it primarily activated dual mechanisms by affecting the soil chemical environment. The Ca(OH)_2_ treatment group was mainly distributed in the negative region of PC1, showing positive correlations with oxidizable fraction of Cd (CdF3) and CdF4, confirming the dominant role of Ca(OH)_2_ in chemical fixation mechanisms. The CaCO_3_ treatment group exhibited a dispersed distribution, demonstrating the diversity of its mechanistic actions, but was primarily associated with stable Cd formation.

In the PCA of plant Cd uptake, PC1 and PC2 explained 57.07% and 21.61% of the total variation, respectively, cumulatively accounting for 78.68% of the variation (Figure 6b). The CaO treatment group showed a strong positive correlation with soil pH, but exhibited negative correlations with available Cd, root Cd, shoot Cd, and grain Cd, reflecting the simultaneous activation of both chemical fixation and ionic competition processes by altering the soil chemical environment. The Ca(OH)_2_ treatment group was primarily positively correlated with available and root Cd, indicating that its chemical fixation effects primarily occurred at the soil–root interface. The CaCO_3_ treatment group was mainly negatively correlated with shoot and grain Cd, confirming that its ionic competition mechanism primarily functioned by blocking translocation processes within plants, which is consistent with its outstanding performance in controlling grain Cd.

## 4. Discussion

### 4.1. Dual Blocking Mechanisms of Lime-Based Materials in Regulating Cd Migration in Acidic Soil–Plant Systems

The meta-analysis results of this study demonstrated that lime-based material application exhibited systematic Cd inhibition effects in acidic soil–plant systems (Figure 1), which primarily manifested as a 33.0% reduction in soil available Cd and significant decreases in Cd content across all plant tissues (grain −44.8%, shoot −33.9%, root −24.6%). Simultaneously, soil physicochemical properties improved (pH increased by 15.2%, CEC increased by 29.5%, and exchangeable Ca increased by 35.2%). This systematic effect resulted from the combined action of soil chemical fixation and plant ionic competition [26]. Soil chemical fixation achieved source control by altering Cd speciation in the soil, whereas plant ionic competition realized terminal blocking by interrupting Cd bioaccumulation processes [27]. These two processes synergistically formed a complete regulatory chain from “source passivation” to “translocation blocking.” Mechanistic control of Cd contamination by lime-based materials primarily involved soil chemical fixation and ionic competition processes to achieve systematic control of Cd pollution from the soil chemical environment to plant physiological uptake.

First, lime-based material application significantly increased the soil pH values, creating alkaline conditions that were favorable for Cd ion precipitation and adsorption. With increasing pH values, Cd ions more readily combined with hydroxide to form Cd hydroxide precipitates, or with carbonate ions to form Cd carbonate precipitates, thereby reducing their concentration in soil solution [28]. The meta-analysis quantified this pH-dependent mechanism: each unit increase in pH corresponded to approximately 21% reduction in available Cd, demonstrating that pH serves as the primary kinetic driver controlling Cd solubility and subsequent bioavailability transformation. Additionally, Ca ion addition enhanced soil CEC, providing more adsorption sites for Cd ions [29]. High Ca ion concentrations could compete with Cd ions for binding sites on soil colloid surfaces while promoting Cd ion migration and transformation into more stable soil components [30]. This adsorption-mediated mechanism is inherently surface-area dependent: lime materials with higher specific surface area (typically Ca(OH)_2_ > CaO > CaCO_3_) provide greater reactive site density, directly correlating with the observed 58.7% available Cd reduction by Ca(OH)_2_ compared to 33.0% overall average. Soil Cd speciation analysis results supported this perspective (Figure 1), with significant increases in the CdF4 (29.5%) and significant decreases in CdF1 (17.5%), indicating that Ca source addition promoted Cd transformation from labile exchangeable forms to relatively stable organic-bound forms [31]. This speciation transformation is an important mechanism for soil Cd passivation. The dominance of chemical fixation over biological processes in controlling Cd removal is evidenced by the stronger correlation between pH changes and available Cd reduction (R^2^ = 0.856) compared to biological factors. This chemical-to-biological cascade determines that soil geochemical transformation serves as the primary rate-limiting step, directly governing plant Cd bioavailability and subsequent ecotoxicological risk through the food chain.

On the other hand, the ionic competition mechanism achieved Cd translocation blocking through competitive binding of Ca and Cd ions at adsorption sites and carrier proteins [32]. The strong negative correlation between exchangeable Ca ions and available Cd (R^2^ = 0.856) quantitatively confirmed the existence of this competitive effect (Figure S1), indicating that the Ca ion concentration was a key factor determining soil Cd bioavailability [6,33]. The ion competition mechanism at plant level was manifested as selective transport by carrier proteins [34]. The linear negative correlation between exchangeable Ca ions and grain Cd (R^2^ = 0.704) established a quantitative relationship between the soil Ca ion concentration and plant Cd accumulation, reflecting the competitive inhibitory effect of Ca ions on Cd ions at carrier proteins. These dual mechanisms are particularly significant for rice production systems, where the grain Cd inhibition rate of 44.8% observed in this meta-analysis directly addresses the primary pathway of human dietary Cd exposure through rice consumption [7]. Overall, lime-based material application synergistically reduced Cd bioavailability and plant accumulation at both soil and plant levels by increasing the soil pH and Ca ion content.

### 4.2. Mechanistic Differences of Three Lime-Based Materials in Cd Immobilization and Control and Long-Term Advantages of Calcium Carbonate

Significant differences in Cd inhibition performance among the three lime-based materials stemmed from their distinct dissolution characteristics and biogeochemical reaction pathways. The dissolution kinetics of lime-based materials constituted the fundamental factor determining their functional characteristics [35]. Ca(OH)_2_ exhibited relatively high solubility (approximately 1.65 g/L), enabling rapid release of substantial quantities of hydroxide and Ca ions, thereby producing immediate and intense alkalinization effects. CaCO_3_ demonstrated lower solubility (approximately 0.014 g/L); however, its dissolution process was regulated by soil carbon dioxide partial pressure, resulting in a slow yet sustained Ca ion release pattern [36,37]. CaO underwent hydration reactions to convert into Ca(OH)_2_, exhibiting intermediate behavior between the other two materials [38,39]. The immediate high-efficiency characteristics of Ca(OH)_2_ were comprehensively demonstrated in the experimental results (Figure 2); i.e., the greatest pH elevation (+27.4%), the most significant reduction in bioavailable Cd (−58.7%), and the strongest root Cd inhibition effect (−35.7%). This intense immediate effect originated from rapid release of hydroxide ions, which can dramatically alter the soil chemical environment within a short period, promoting Cd hydroxide precipitation formation while simultaneously activating Cd adsorption sites on iron–manganese oxide surfaces [4]. This rapid geochemical immobilization directly translates to reduced bioavailability, as evidenced by the 58.7% reduction in available Cd, which mechanistically prevents root uptake and limits trophic transfer to higher organisms, thereby mitigating ecotoxicological effects across the soil–plant–consumer continuum. However, such intense environmental chemical changes may also impose stress on soil ecosystems.

In contrast, although CaCO_3_ exhibited relatively moderate immediate effects (Figure 2a), it demonstrated the optimal performance in controlling grain Cd (−49.6%), which is crucial for rice where grain Cd accumulation poses the most severe food safety risk, reflecting its unique long-term advantages. This phenomenon stemmed from the self-adaptive response characteristics of CaCO_3_ to soil microenvironmental changes [40]. The crystalline structure of CaCO_3_ (calcite polymorph with rhombohedral lattice) exhibits lower dissolution enthalpy compared to amorphous Ca(OH)_2_, resulting in more gradual but sustained Ca^2+^ release kinetics that maintain optimal soil solution chemistry throughout extended crop growth periods, as reflected in its superior grain Cd control (−49.6%). CaCO_3_ underwent selective dissolution under rhizosphere acidic conditions, enabling dynamic regulation of Ca ion release rates according to plant requirements and soil conditions, thereby providing continuous and stable Ca ion supply throughout the entire crop growth period, particularly during the critical grain development stage [41,42]. This sustained supply of Ca ions was particularly crucial during grain filling, maintaining the competitive inhibition state of carrier proteins and effectively blocking long-distance Cd transport from phloem to grains [43], thus achieving long-term effective control of grain Cd accumulation.

Dose–response relationship analysis revealed distinct dose-dependent characteristics among different lime-based materials. CaO exhibited the strongest dose-dependent relationship (Figure 4), with grain Cd inhibition effects significantly enhanced as the theoretical values of Ca ions increased (R^2^ = 0.310, *p* < 0.001). Although CaCO_3_ showed relatively weak dose dependency (grain Cd: R^2^ = 0.210, *p* < 0.001), it demonstrated strong inhibition trends for soil bioavailable Cd under high-dose conditions (R^2^ = 0.570, *p* < 0.05). Notably, the dose effect of Ca(OH)_2_ was relatively inconspicuous, which may have been related to its rapid dissolution characteristics and immediate action mechanism [21,44]. Unlike previous studies that attributed CaCO_3_ efficacy primarily to pH buffering [16,17], our dose–response analysis reveals that sustained Ca^2+^ release—not merely pH stabilization—drives its superior grain Cd control, as evidenced by the strong negative correlation between exchangeable Ca and grain Cd (R^2^ = 0.704) persisting across diverse pH ranges. Subgroup analysis in this study revealed that when the theoretical values for Ca ions ranged from 500–1000 kg/ha (Figure 2), the reduction in soil bioavailable Cd was relatively substantial (−43.1%). When the theoretical values for Ca ions exceeded 1000 kg/ha, the reduction in grain Cd reached its maximum (−51.4%), a trend consistent with regression analysis results for CaO and CaCO_3_, indicating the existence of dominant process transitions under different dosage conditions.

This study found that Ca(OH)_2_ may be more suitable for situations requiring rapid soil Cd passivation, and that CaCO_3_ represents a more appropriate choice for long-term grain Cd prevention and control strategies. From a dose-effect perspective, both CaO and CaCO_3_ exhibited obvious dose dependency, whereas Ca(OH)_2_ effects remained relatively stable without significant influence from dosage variations. These findings provide an important reference for lime-based material selection and dosage determination for different application scenarios.

### 4.3. Mechanistic Analysis and Economic Feasibility Evaluation of Passivation Performance of Calcium Carbonate on Cd

Across all dosage ranges, CaCO_3_ demonstrated excellent control effects on grain Cd (Figure 5), particularly under high Ca ion theoretical value (>1000 kg/ha) conditions where grain Cd inhibition reached −65.7%, and was significantly superior to the other two lime-based materials. Primary pathways for Cd passivation by CaCO_3_ included carbonate precipitation and solid solution formation [45]. Carbonate ions released from CaCO_3_ dissolution can form Cd carbonate precipitates with Cd ions (Ksp = 1.8 × 10^−14^), and Cd ions can also substitute for Ca ions to enter calcite crystal lattices, forming Ca_1−x_Cd_x_CO_3_ solid solution structures [40]. Due to the similar ionic radii of Cd ions (0.97 Å) and Ca ions (1.06 Å), Cd ions can partially occupy Ca ion positions in calcite lattices through isomorphous substitution, forming thermodynamically extremely stable solid solutions [46]. This lattice substitution not only significantly reduces Cd chemical activity but, more importantly, achieves long-term geochemical stability of Cd. Even when acidification or redox conditions change [47], lattice-incorporated Cd remains extremely difficult to reactivate.

The continuous release of Ca ions from CaCO_3_ ensures its stable effects under different application dosages. Unlike rapidly dissolving Ca(OH)_2_, CaCO_3_ possesses unique environmentally responsive dissolution characteristics: dissolution rates increase under acidic soil conditions to provide more Ca ions, and decrease under alkaline conditions to avoid excessive alkalization [48]. This self-regulatory mechanism ensures a dynamic equilibrium between Ca ion release and the soil environment [42,49]. More importantly, organic acids such as citric and malic acids secreted by plant roots can form localized acidification microzones in the rhizosphere, precisely regulating selective CaCO_3_ dissolution around root systems, achieving spatiotemporal precision matching of the Ca ion supply [14,43]. This rhizosphere-regulated continuous Ca ion supply mechanism ensures maintenance of effective ionic competition during critical periods of plant Cd uptake, particularly providing sustained protection during sensitive grain development stages [50,51]. This represents the core reason for the sustained advantages of CaCO_3_ in controlling grain Cd. Furthermore, it should be acknowledged that the efficacy of this ionic competition mechanism might be modulated by soil texture (e.g., clay vs. sand content), which dictates the soil’s cation exchange capacity and buffering potential, representing a critical variable to be explored in future investigations.

From an economic benefit perspective, CaCO_3_ demonstrated the optimal cost-effectiveness ratio for controlling grain Cd. Our research showed that at application rates > 2 t/ha, CaCO_3_, Ca(OH)_2_, and CaO significantly reduced the grain Cd content by 60.9%, 57.3%, and 45.8%, respectively (Figure 5). Based on international market pricing (CaCO_3_ production costs of 100–200 yuan (RMB)/ton, CaO at 400–600 yuan/t, and Ca(OH)_2_ at 700–1000 yuan/ton), in severely Cd-contaminated areas, the cost of CaCO_3_ application per 1% reduction in grain Cd is 1.6–3.3 yuan; whereas, CaO and Ca(OH)_2_ application costs are significantly higher, at 8.7–13.1 yuan and 12.2–17.4 yuan, respectively [35,52]. Crucially, the economic advantage of CaCO_3_ extends beyond its lower unit price. Unlike CaO and Ca(OH)_2_, which react rapidly and may necessitate more frequent re-application due to potential soil re-acidification, CaCO_3_ functions as a slow-release agent that maintains long-term geochemical stability. This sustained-release characteristic effectively extends the remediation interval, thereby significantly reducing cumulative labor costs and mechanical expenses over multiple cropping seasons. Additionally, CaCO_3_ production processes have relatively low energy consumption and superior environmental friendliness [53]. Therefore, comprehensively considering remediation effectiveness, economic costs, and environmental impacts, CaCO_3_ demonstrated optimal overall advantages in Cd-contaminated farmland remediation and is particularly suitable for large-scale applications.

## 5. Conclusions

This study systematically elucidated the dual mechanisms by which lime-based materials regulate Cd migration in acidic soil–plant systems through a meta-analysis, specifically addressing the synergistic effects of chemical fixation and ionic competition. The results demonstrated that lime-based material application significantly reduced soil bioavailable Cd (33.0%) and grain Cd accumulation (44.8%), while simultaneously increasing soil pH by an average of 15.6% and exchangeable Ca by 35.2%. This systematic Cd inhibition effect stemmed from the transformation of Cd speciation from mobile to stable forms (residual Cd increased by 29.5% and weak acid-extractable Cd decreased by 17.5%), and the strong ionic competition relationship between exchangeable Ca ions and grain Cd (R^2^ = 0.704). Importantly, this study revealed differentiated action modes of three major lime-based materials within the dual mechanism framework: Ca(OH)_2_ primarily functions through rapid chemical fixation mechanisms, resulting in the most significant reduction in soil bioavailable Cd (58.7%) and strong inhibition effects on root Cd (35.7%); CaO exhibited pronounced application dose-dependent characteristics, with grain Cd inhibition effects significantly enhanced as application rates increased (R^2^ = 0.31), and; CaCO_3_ demonstrated optimal performance in controlling grain Cd through continuous ionic competition processes, in particular achieving inhibition rates up to 65.7% under high application doses. These findings expand the theoretical understanding of lime-based passivation technology, elevating the mechanistic knowledge from simple pH regulation to a systematic framework of synergistic regulation through chemical fixation and ionic competition. The established theoretical framework not only provides new scientific foundations for soil heavy metal remediation but also establishes the basis for precision material-selection strategies based on dissolution kinetics, particularly highlighting the advantages of CaCO_3_ with regard to comprehensive performance and economic benefits. For rice production systems specifically, where grain Cd accumulation represents the primary human exposure pathway, CaCO_3_’s sustained ionic competition mechanism offers optimal long-term grain Cd control with economic viability. This research provides scientific support for standardized application of Cd-contaminated farmland remediation technologies.

## Figures and Tables

**Table 1:** Comparison between this study and previous meta-analyses of lime-based materials for cadmium remediation.

Reference	Publications Reviewed	Lime-Based Materials Specificity	Calcium-Related Indicators	Plant Cadmium Distribution	Cadmium Speciation Analysis
Kong et al. [16]	39 studies (rice only)	Calcium carbonate, calcium hydroxide, calcium oxide (mixed analysis)	Not included	Grain cadmium only	Not included
Liao et al. [21]	35 studies (rice only)	Lime-based materials (undifferentiated)	Not included	Grain cadmium and yield	Not included
He et al. [17]	30 studies	Carbon carbonate, calcium carbonate, calcium hydroxide, calcium oxide (grouped)	Not included	Shoot and grain cadmium	Not included
This study	55 studies (rice focus)	Carbon carbonate, calcium hydroxide, carbon oxide (separate analysis)	Soil exchangeable calcium ions, grain calcium	Root, shoot, grain, husk cadmium	CdF1–F4 fractions

## Data Availability

Data will be provided as requested.

## Supplementary Materials

The following supporting information can be downloaded at: https://www.mdpi.com/article/10.3390/biology15030207/s1, Figure S1: Relationships between ln(*RR*) of exchangeable calcium and (a) available cadmium, (b) grain cadmium, (c) grain calcium; ln(*RR*) of root cadmium and (d) grain cadmium, (e) stem cadmium; (f) ln(*RR*) of stem cadmium and grain cadmium; Figure S2: Relationships between ln(*RR*) of soil pH and ln(*RR*) of exchangeable calcium (a), available cadmium (b), soil cation exchange capacity (c), grain cadmium (d), root cadmium (e) and stem cadmium (f). Figure S3: The weighted effect size of lime materials in soil under different subgroups. Vertical error bars indicate 95% confidence intervals. Figure S4: Relationships between the theoretical value for Ca^2+^ of lime materials and ln(*RR*) of soil available cadmium, soi exchangeable calcium, Grain Ca, Grain Cd, Root Cd, Stem Cd. Table S1: Results of publication bias about datasets in this study. “N” is the number of observations. Text S1. List of all articles included in this meta-analysis [54,55,56,57,58,59,60,61,62,63,64,65,66,67,68,69,70,71,72,73,74,75,76,77,78,79,80,81,82,83,84,85,86,87,88,89,90,91,92,93,94,95,96,97,98,99,100,101,102,103,104,105,106,107,108].
