# Supplementary material for: Mechanistic Pathways Controlling Cadmium Bioavailability and Ecotoxicity in Agricultural Systems: A Global Meta-Analysis of Lime Amendment Strategies
Source: Biology (Basel). 2026 Jan 23;15(3):207. doi: 10.3390/biology15030207 (PMC12896412; doi:10.3390/biology15030207)
Supplement: Supplementary file 1 [file biology-15-00207-s001.zip › Figure.S1.pdf]

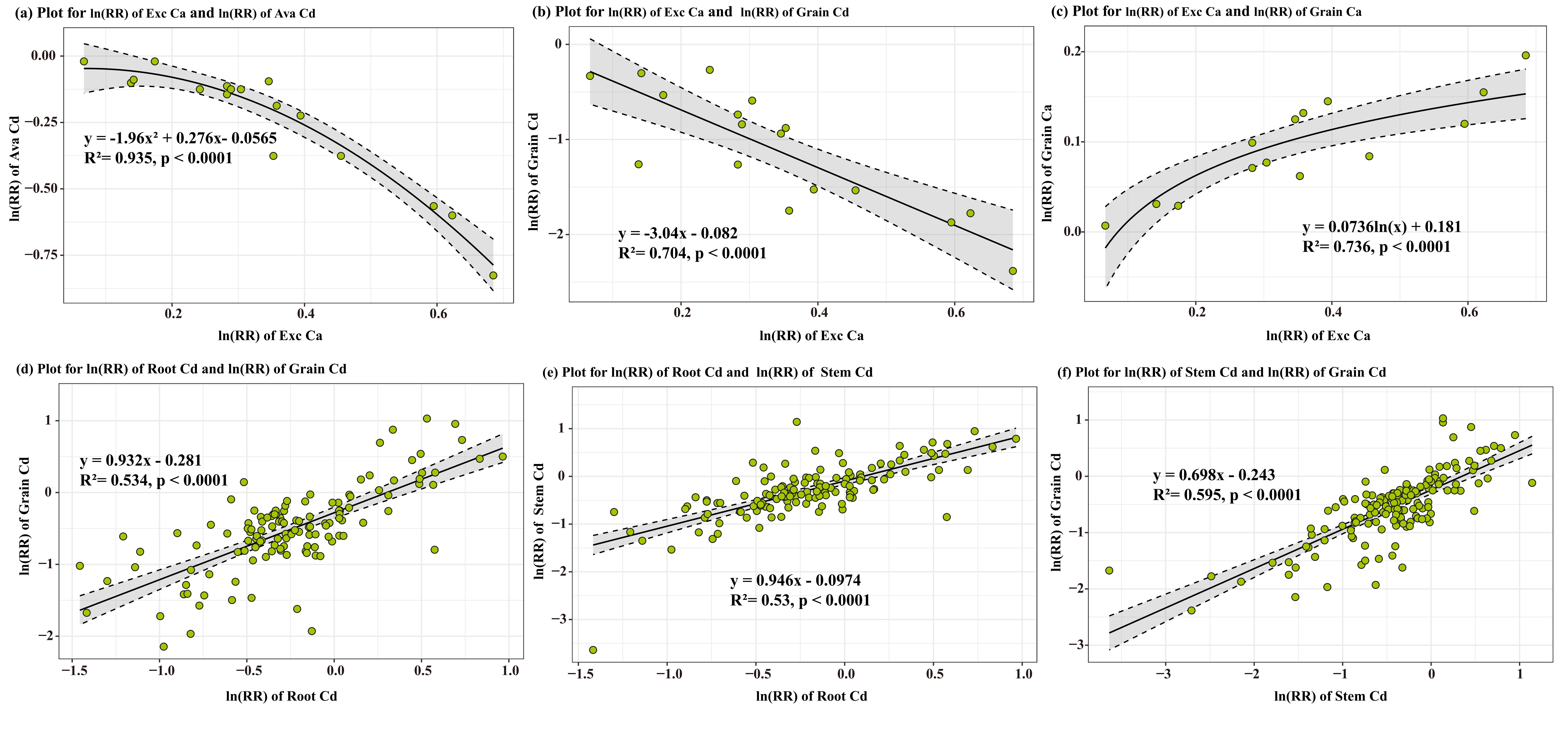
(a) Plot for ln(RR) of Exc Ca and ln(RR) of Ava Cd
y = -1.96x² + 0.276x- 0.0565
R²= 0.935, p < 0.0001
ln(RR) of Ava Cd
ln(RR) of Exc Ca
(b) Plot for ln(RR) of Exc Ca and ln(RR) of Grain Cd
y = -3.04x - 0.082
R²= 0.704, p < 0.0001
ln(RR) of Grain Cd
ln(RR) of Exc Ca
(c) Plot for ln(RR) of Exc Ca and ln(RR) of Grain Ca
y = 0.0736ln(x) + 0.181
R²= 0.736, p < 0.0001
ln(RR) of Grain Ca
ln(RR) of Exc Ca
(d) Plot for ln(RR) of Root Cd and ln(RR) of Grain Cd
y = 0.932x - 0.281
R²= 0.534, p < 0.0001
ln(RR) of Grain Cd
ln(RR) of Root Cd
(e) Plot for ln(RR) of Root Cd and ln(RR) of Stem Cd
y = 0.946x - 0.0974
R²= 0.53, p < 0.0001
ln(RR) of Stem Cd
ln(RR) of Root Cd
(f) Plot for ln(RR) of Stem Cd and ln(RR) of Grain Cd
y = 0.698x - 0.243
R²= 0.595, p < 0.0001
ln(RR) of Grain Cd
ln(RR) of Stem Cd
