# Supplementary material for: Mechanistic Pathways Controlling Cadmium Bioavailability and Ecotoxicity in Agricultural Systems: A Global Meta-Analysis of Lime Amendment Strategies
Source: Biology (Basel). 2026 Jan 23;15(3):207. doi: 10.3390/biology15030207 (PMC12896412; doi:10.3390/biology15030207)
Supplement: Supplementary file 1 [file biology-15-00207-s001.zip › Figure.S2.pdf]

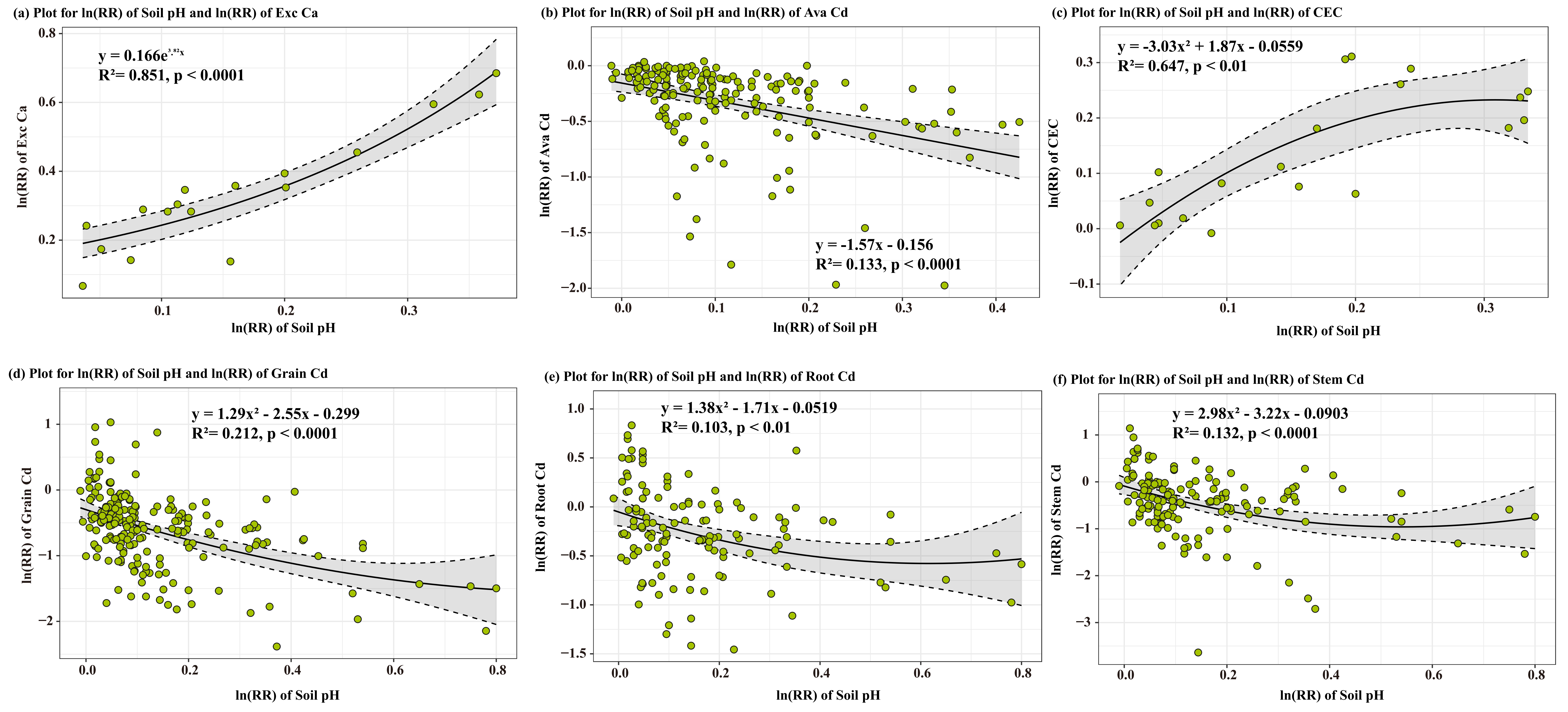

(a) Plot for ln(RR) of Soil pH and ln(RR) of Exc Ca
y = 0.166e^3.82x
R²= 0.851, p < 0.0001
ln(RR) of Exc Ca
ln(RR) of Soil pH
0.8
0.6
0.4
0.2
0.1
0.2
0.3
(b) Plot for ln(RR) of Soil pH and ln(RR) of Ava Cd
y = -1.57x - 0.156
R²= 0.133, p < 0.0001
ln(RR) of Ava Cd
ln(RR) of Soil pH
0.0
−0.5
−1.0
−1.5
−2.0
0.0
0.1
0.2
0.3
0.4
(c) Plot for ln(RR) of Soil pH and ln(RR) of CEC
y = -3.03x² + 1.87x - 0.0559
R²= 0.647, p < 0.01
ln(RR) of CEC
ln(RR) of Soil pH
0.3
0.2
0.1
0.0
−0.1
0.1
0.2
0.3
(d) Plot for ln(RR) of Soil pH and ln(RR) of Grain Cd
y = 1.29x² - 2.55x - 0.299
R²= 0.212, p < 0.0001
ln(RR) of Grain Cd
ln(RR) of Soil pH
1
0
−1
−2
0.0
0.2
0.4
0.6
0.8
(e) Plot for ln(RR) of Soil pH and ln(RR) of Root Cd
y = 1.38x² - 1.71x - 0.0519
R²= 0.103, p < 0.01
ln(RR) of Root Cd
ln(RR) of Soil pH
1.0
0.5
0.0
−0.5
−1.0
−1.5
0.0
0.2
0.4
0.6
0.8
(f) Plot for ln(RR) of Soil pH and ln(RR) of Stem Cd
y = 2.98x² - 3.22x - 0.0903
R²= 0.132, p < 0.0001
ln(RR) of Stem Cd
ln(RR) of Soil pH
1
0
−1
−2
−3
0.0
0.2
0.4
0.6
0.8
