# Supplementary material for: Mechanistic Pathways Controlling Cadmium Bioavailability and Ecotoxicity in Agricultural Systems: A Global Meta-Analysis of Lime Amendment Strategies
Source: Biology (Basel). 2026 Jan 23;15(3):207. doi: 10.3390/biology15030207 (PMC12896412; doi:10.3390/biology15030207)
Supplement: Supplementary file 1 [file biology-15-00207-s001.zip › Figure.S4.pdf]

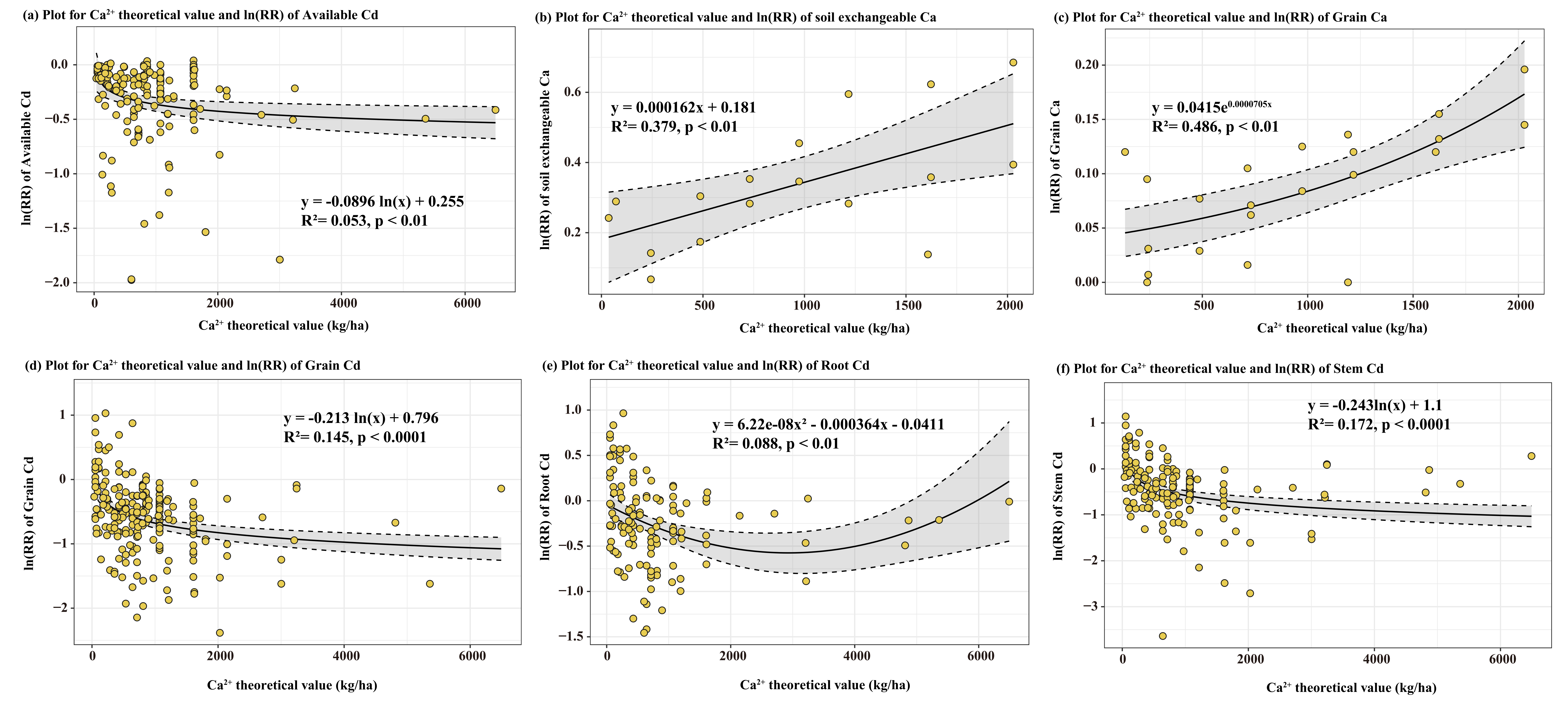
(a) Plot for Ca²⁺ theoretical value and ln(RR) of Available Cd
y = -0.0896 ln(x) + 0.255
R²= 0.053, p < 0.01
ln(RR) of Available Cd
Ca²⁺ theoretical value (kg/ha)
(b) Plot for Ca²⁺ theoretical value and ln(RR) of soil exchangeable Ca
y = 0.000162x + 0.181
R²= 0.379, p < 0.01
ln(RR) of soil exchangeable Ca
Ca²⁺ theoretical value (kg/ha)
(c) Plot for Ca²⁺ theoretical value and ln(RR) of Grain Ca
y = 0.0415e^0.0000705x
R²= 0.486, p < 0.01
ln(RR) of Grain Ca
Ca²⁺ theoretical value (kg/ha)
(d) Plot for Ca²⁺ theoretical value and ln(RR) of Grain Cd
y = -0.213 ln(x) + 0.796
R²= 0.145, p < 0.0001
ln(RR) of Grain Cd
Ca²⁺ theoretical value (kg/ha)
(e) Plot for Ca²⁺ theoretical value and ln(RR) of Root Cd
y = 6.22e-08x² - 0.000364x - 0.0411
R²= 0.088, p < 0.01
ln(RR) of Root Cd
Ca²⁺ theoretical value (kg/ha)
(f) Plot for Ca²⁺ theoretical value and ln(RR) of Stem Cd
y = -0.243ln(x) + 1.1
R²= 0.172, p < 0.0001
ln(RR) of Stem Cd
Ca²⁺ theoretical value (kg/ha)
