# Supplementary material for: Mechanistic Pathways Controlling Cadmium Bioavailability and Ecotoxicity in Agricultural Systems: A Global Meta-Analysis of Lime Amendment Strategies
Source: Biology (Basel). 2026 Jan 23;15(3):207. doi: 10.3390/biology15030207 (PMC12896412; doi:10.3390/biology15030207)
Supplement: Supplementary file 1 [file biology-15-00207-s001.zip › Supplementary materials.pdf]

Mechanistic Pathways Controlling Cadmium Bioavailability and Ecotoxicity in Agricultural Systems: A Global Meta-Analysis of Lime Amendment Strategies

Jianxun Qin [1,2,3,4], Keke Sun [4], Yongfeng Sun [4], Shunting He [4], Yanwen Zhao [4], Junyuan Qi [4], Yimin Lan [4], Beilei Wei [4,*] and Ziting Wang [4,*]

[1] School of the Earth Sciences and Resources, China University of Geosciences, Beijing 100083, China; qinjianxun@gxdzdcy.cn
[2] Guangxi Institute of Geological Survey, Nanning 530023, China
[3] Guangxi Engineering Research Center for Medical Geology, Nanning 530023, China
[4] State Key Laboratory for Conservation and Utilization of Subtropical Agri-Biological Resources, Guangxi Key Laboratory for Sugarcane Biology, College of Agriculture, Guangxi University, Nanning 530004, China; sun1967230965@163.com (K.S.); 18778977421@163.com (Y.S.); shuntinghe0319@163.com (S.H.); 15678585108@163.com (Y.Z.); 17351995197@163.com (J.Q.); 2517300022@st.gxu.edu.cn (Y.L.)
* Correspondence: weibeilei159@163.com (B.W.); zitingwang@gxu.edu.cn (Z.W.)

Tables

Table S1 Results of publication bias about datasets in this study. “N” is the number of observations.

Factors	Observations sizes (N)	5N+10	Fail-safe number	Publication bias
pH	235	1185	1761141	NO
SOM	23	125	100	YES
CEC	20	110	23748	NO
Exc Ca	17	95	900	NO
ST Cd	29	155	88	YES
CdF1	57	295	7576	NO
CdF2	57	295	494	NO
CdF3	57	295	8	YES
CdF4	57	295	11	YES
Cd_R	142	720	133824	NO
Cd_S	172	870	102177	NO
Cd_H	33	175	5563	NO
Cd_G	232	1170	1021233	NO
Yield	130	660	261570106	NO
Ava_Cd	188	950	832584	NO

Figure S1

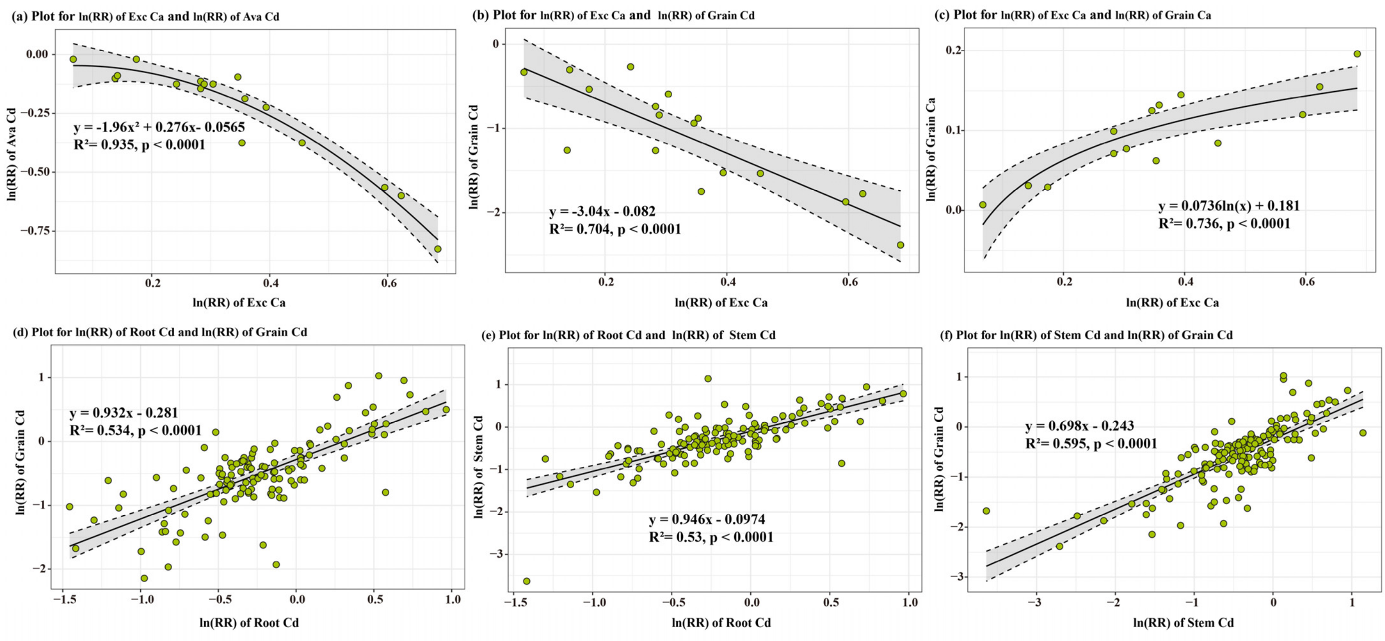

Figure S1 Relationships between ln(*RR*) of exchangeable calcium and (a) available cadmium, (b) grain cadmium, (c) grain calcium; ln(*RR*) of root cadmium and (d) grain cadmium, (e) stem cadmium; (f) ln(*RR*) of stem cadmium and grain cadmium.

Figure S2

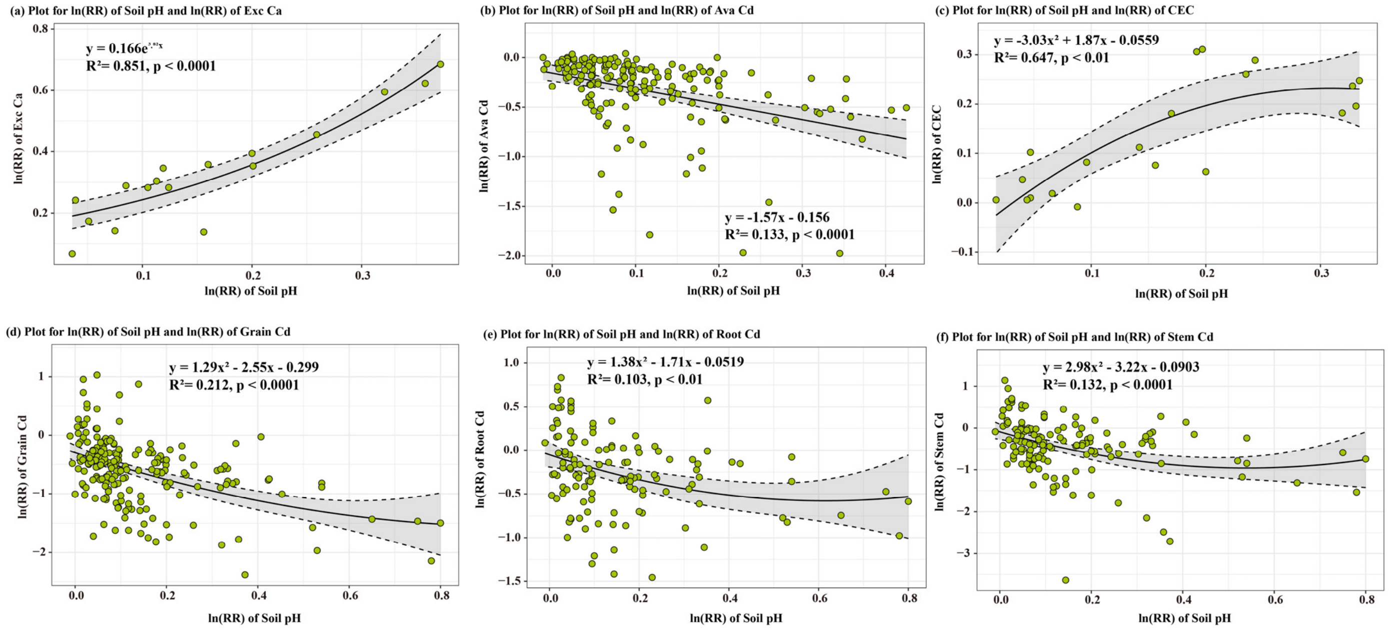

Figure S2 Relationships between ln(*RR*) of soil pH and ln(*RR*) of exchangeable calcium (a), available cadmium (b), soil cation exchange capacity (c), grain cadmium (d), root cadmium (e) and stem cadmium (f).

Figure S3

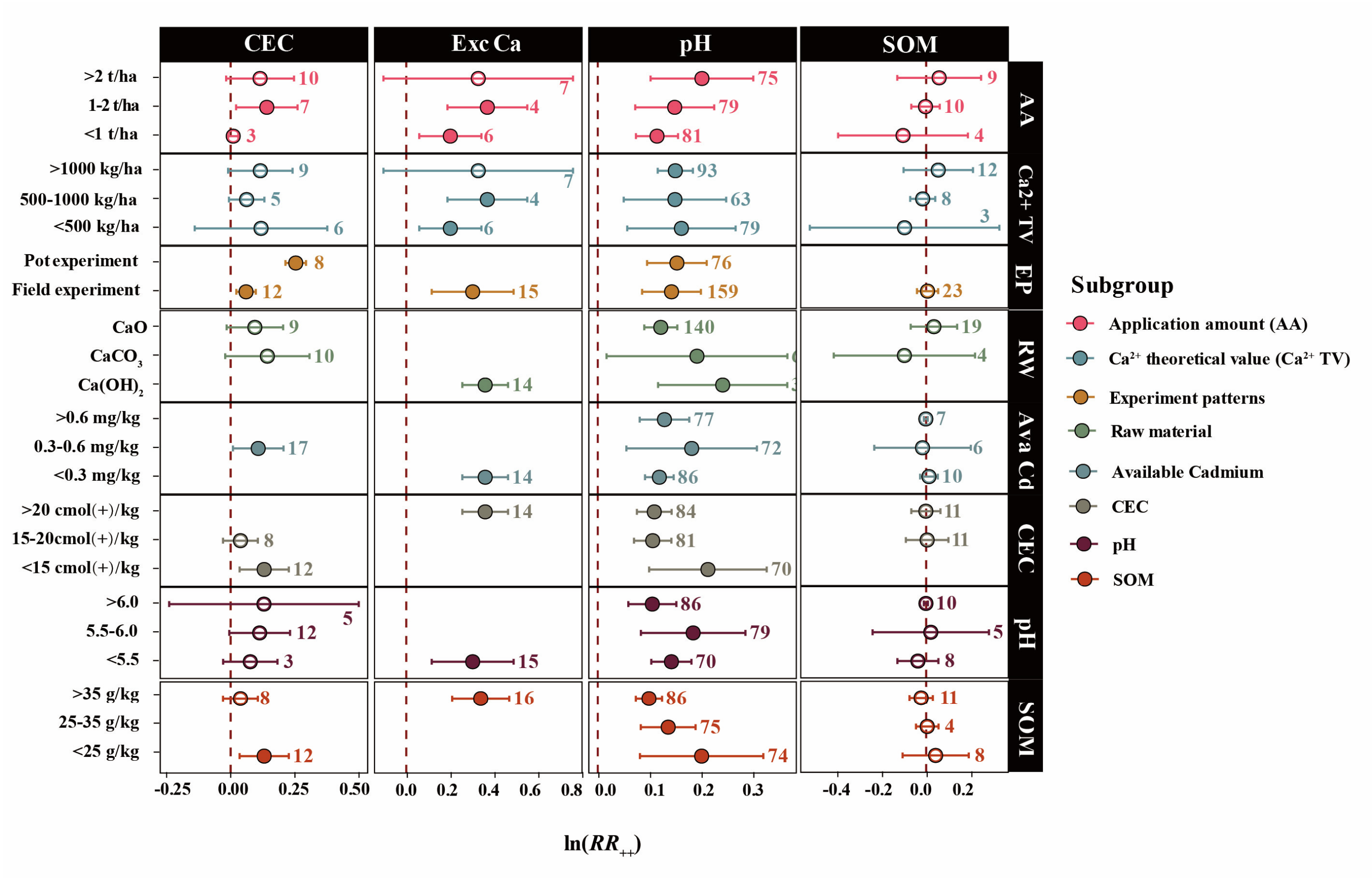

Figure S3 The weighted effect size of lime materials in soil under different subgroups. Vertical error bars indicate 95% confidence intervals. The Arabic numerals represent the sample size of the response variables. CEC, soil cation exchange capacity; Exc Ca, soil exchangeable calcium content; SOM, soil organic matter.

Figure S4

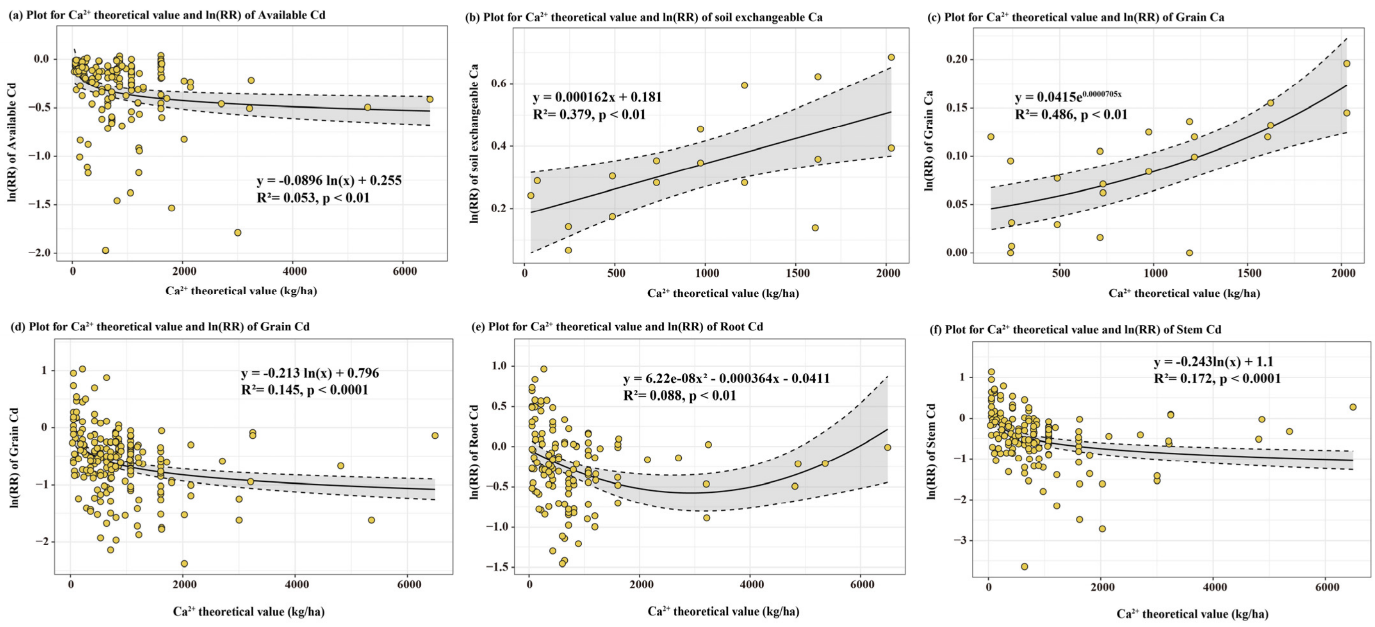

Figure S4 Relationships between the theoretical value for Ca^{2+} of lime materials and ln(*RR*) of soil available cadmium, soi exchangeable calcium, Grain Ca, Grain Cd, Root Cd, Stem Cd.

Text S1 List of all articles included in this meta-analysis**.**

Bian, R., Li, L., Bao, D., et al. 2016. Cd immobilization in a contaminated rice paddy by inorganic stabilizers of calcium hydroxide and silicon slag and by organic stabilizer of biochar. Environmental Science and Pollution Research 23: 10028-10036. https://doi.org/10.1007/s11356-016-6214-3

Cao, S., Zhou, W., Zhou, Y., et al. 2017. The Cadmium Reduction Effect of Silicon Calcium Magnesium Soil Conditioner on Acid Cadmium Polluted Soil and Rice. Journal of Henan Agricultural Sciences 46 (12).

Chen, H., Zhang, W., Yang, X., et al. 2018. Effective methods to reduce cadmium accumulation in rice grain. Chemosphere 207: 699-707. https://doi.org/10.1016/j.chemosphere.2018.05.143

Chen, H., Wang, P., Chang, J., et al. 2021. Producing Cd-safe rice grains in moderately and seriously Cd-contaminated paddy soils. Chemosphere 267: 128893. https://doi.org/10.1016/j.chemosphere.2020.128893

Cheng, Z., Shi, J., He, Y., et al. 2022. Assembly of root-associated bacterial community in cadmium contaminated soil following five-year consecutive application of soil amendments: Evidences for improved soil health. Journal of Hazardous Materials 426: 128095. https://doi.org/10.1016/j.jhazmat.2021.128095

Dai, Y., Yang, Y., Fu, K., et al. 2021. Effects and application risk of liming on cadmium uptake by rice. Chinese Journal of Environmental Engineering 15 (04).

Dong, H., Tang, S., Ye, S., et al. 2016. Effect of lime on the transfer of Cd and Pb in the soil-rice cultivation system and their accumulation in the rice grains. Journal of Safety and Environment 16 (02).

Duan, M., Wang, S., Huang, D., et al. 2018. Effectiveness of simultaneous applications of lime and zinc/iron foliar sprays to minimize cadmium accumulation in rice. Ecotoxicology and Environmental Safety 165: 510-515. https://doi.org/10.1016/j.ecoenv.2018.09.037

Gong, L., Chen, K., Li, D., et al. Remediation effects of mixed amendment at different application levels on

cadmium-contaminated farmland soil. Journal of Zhejiang University (Agriculture and Life Sciences) 48 (3): 359-368. https://doi.org/10.3785/j.issn.1008-9209.2021.05.172

Guo, F., Ding, C., Zhou, Z., et al. 2018. Effects of combined amendments on crop yield and cadmium uptake in two cadmium contaminated soils under rice-wheat rotation. Ecotoxicology and Environmental Safety 148: 303-310. https://doi.org/10.1016/j.ecoenv.2017.10.043

Hamid, Y., Liu, L., Usman, M., et al. 2022. Organic/inorganic amendments for the remediation of a red paddy soil artificially contaminated with different cadmium levels: Leaching, speciation, and phytoavailability tests. Journal of Environmental Management 303: 114148. https://doi.org/10.1016/j.jenvman.2021.114148

Hamis, Y., Tang, L., Hussain, B., et al. 2020. Efficiency of lime, biochar, Fe containing biochar and composite amendments for Cd and Pb immobilization in a co-contaminated alluvial soil. Environmental Pollution 257: 113609. https://doi.org/10.1016/j.envpol.2019.113609

He, Y., Huang, D., Zhu, Q., et al. 2017. A three-season field study on the in-situ remediation of Cd-contaminated paddy soil using lime, two industrial by-products, and a low-Cd-accumulation rice cultivar. Ecotoxicology and Environmental Safety 136: 135-141. https://doi.org/10.1016/j.ecoenv.2016.11.005

Hu, H., Gao, L., Zhang, H., et al. 2022. Effectiveness of Passivator Amendments and Optimized Fertilization for Ensuring the Food Safety of Rice and Wheat from Cadmium-Contaminated Farmland. Sustainability 14 (22), 15026. https://doi.org/10.3390/su142215026

Huang, B., Wu, Q., Xiao, H., et al. 2020. Effects of continuous application of lime for three years on cadmium concentration and uptake by wheat and rice in Cd contaminated soil. Soil and Fertilizer Sciences in China 03: 138-143.

Huang, G., Ding, C., Hu, Z., et al. 2018. Topdressing iron fertilizer coupled with pre-immobilization in acidic paddy fields reduced cadmium uptake by rice (Oryza sativa L.). Science of The Total

Environment 636: 1040-1047. https://doi.org/10.1016/j.scitotenv.2018.04.369

Huang, G., Ding, C., Guo, N., et al. 2021. Polymer-coated manganese fertilizer and its combination with lime reduces cadmium accumulation in brown rice (Oryza sativa L.). Journal of Hazardous Materials 415: 125597. https://doi.org/10.1016/j.jhazmat.2021.125597

Huang, Y., Sheng, H., Zhou, P., et al. 2020. Remediation of Cd-contaminated acidic paddy fields with four-year consecutive liming. Ecotoxicology and Environmental Safety 188: 109903. https://doi.org/10.1016/j.ecoenv.2019.109903

Huang, Y., Liao, M., Ye, Z., et al. 2017. Cd Concentrations in Two Low Cd Accumulating Varieties of Rice and Their Relationships With Soil Cd Content and Their Regulation Under Field Conditions. Journal of Ecology and Rural Environment 33 (8): 748-754. https://dx.doi.org/10.11934/j.issn.1673-4831.2017.08.011

Hamid, Y., Tang, L., Lu, M., et al. 2019. Assessing the immobilization efficiency of organic and inorganic amendments for cadmium phytoavailability to wheat. Journal of Soil and Sediments 19: 3708-3717. https://doi.org/10.1007/s11368-019-02344-0

Hamid, Y., Tang, L., Wang, X., et al. 2018. Immobilization of cadmium and lead in contaminated paddy field using inorganic and organic additives. Scientific Reports 8, 17839. https://doi.org/10.1038/s41598-018-35881-8

Li, B., Yang, L., Wang, C., et al. 2018. Effects of organic-inorganic amendments on the cadmium fraction in soil and its accumulation in rice (Oryza sativa L.). Environmental Science and Pollution Research International 26 (14): 13762-13772. https://doi.org/10.1007/s11356-018-2914-1

Li, D., Liu, H., Gao, M., et al. 2022. Effects of soil amendments, foliar sprayings of silicon and selenium and their combinations on the reduction of cadmium accumulation in rice. Pedosphere 32 (4): 649-659. https://doi.org/10.1016/S1002-0160(21)60052-8

Li, F., Ai, S., Wang, Y., et al. 2016. In Situ Field-Scale Remediation of Low Cd-Contaminated Paddy Soil

Using Soil Amendments. Water, Air, & Soil Pollution 227, 342. https://doi.org/10.1007/s11270-016-3041-6

Li, G., Cheng, Q., Cheng, H. 2021. Remediation of Cd Contaminated Acidic Rice Fields Using the Combined Application of Lime and Organic Matter. Environmental Science 42(2): 925-931. https://doi.org/10.13227/j.hjkx.202007237

Li, P., Wang, X., Zhang, T., et al. 2009. Distribution and Accumulation of Copper and Cadmium in Soil–Rice System as Affected by Soil Amendments. Water, Air, and Soil Pollution 196: 29-40. https://doi.org/10.1007/s11270-008-9755-3

Li, X., Peng, P., Chen, Q., et al. 2018. Evaluation of Calcium Oxide of Quicklime and Si–Ca–Mg Fertilizer for Remediation of Cd Uptake in Rice Plants and Cd Mobilization in Two Typical Cd-Polluted Paddy Soils. International of Environmental Research 12: 877-885. https://doi.org/10.1007/s41742-018-0142-7

Li, X., Yang, C., Liu, Y., et al. 2021. EFFECT OF PASSIVATORS ON Cd AVAILABILITY IN FARMLAND SOIL AND Cd UPTAKE BY DIFFERENT RICE VARIETIES. Environmental Engineering 39 (9): 211-216. http://dx.doi.org/10.13205/j.hjgc.202109030

Liu, B., Ji, X., Peng, H., et al. 2012. Effects of phosphorous fertilizers on phytoavailability of cadmium in its contaminated soil and related mechanisms. Chinese Journal of Applied Ecology 23 (6): 1585-1590.

Liu, Z., Huang, Y., Ji, X., et al. 2020. Effects and Mechanism of Continuous Liming on Cadmium Immobilization and Uptake by Rice Grown on Acid Paddy Soils. Journal of Soil Science and Plant Nutrition 20: 2316-2328. https://doi.org/10.1007/s42729-020-00297-9

Lu, J., Gong, L., Cai, M., et al. 2022. Study on the Passivation Effect of Minerals on Cd in Farmland Soil with Light Heavy Metal Pollution. Journal of Ecology and Rural Environment 38 (3): 391-398. https://dx.doi.org/10.19741/j.issn.1673-4831.2021.0148

Luo, W., Yang, S., Khan, M.A., et al. 2020. Mitigation of Cd accumulation in rice with water management

and calcium-magnesium phosphate fertilizer in field environment. Environmental Geochemistry and Health 42: 3877-3886. https://doi.org/10.1007/s10653-020-00648-6

Meng, L., Huang, T., Shi, J., et al. 2018. Decreasing cadmium uptake of rice (Oryza sativa L.) in the cadmium-contaminated paddy field through different cultivars coupling with appropriate soil amendments. Journal of Soils and Sediments 19: 1788-1798. https://doi.org/10.1007/s11368-018-2186-x

Shi, L., Guo, Z., Liang, F., et al. 2017. Effects of lime and water management on uptake and translocation of cadmium in rice. Transactions of the Chinese Society of Agricultural Engineering 33 (7): 111-117.

Shi, L., Guo, Z., Liu, S., et al. 2021. Effects of combined soil amendments on Cd accumulation, translocation and food safety in rice: a field study in southern China. Environmental Geochemistry and Health 44: 2451-2463. https://doi.org/10.1007/s10653-021-01033-7

Shi, L., Guo, Z., Peng, C., et al. 2018. Lime based amendments inhibiting uptake of cadmium in rice planted in contaminated soils. Transactions of the Chinese Society of Agricultural Engineering 34 (11): 209-216. https://dx.doi.org/10.11975/j.issn.1002-6819.2018.11.027

Shi, L., Guo, Z., Peng, C., et al. 2019. Immobilization of cadmium and improvement of bacterial community in contaminated soil following a continuous amendment with lime mixed with fertilizers: A four-season field experiment. Ecotoxicology and Environmental Safety 171: 425-434. https://doi.org/10.1016/j.ecoenv.2019.01.006

Tang, X., Zhou, X., Huang, F., et al. 2020. Study on the Effect of Comprehensive Treatment Technology on Rice Cadmium Content Under Different Cd Polluted Cultivated Land. Journal of Ecology and Rural Environment 36 (10): 1339-1346. https://dx.doi.org/10.19741/j.issn.1673-4831.2020.0232

Tian, F., Ji, X., Xie, Y., et al. 2016. Alkaline slow-release fertilizer decreased rice Cd uptake at Cd-contaminated paddy fields. Journal of Agro-Environment Science 35 (11).

Wang, X., Zhang, D., Zhao, S., et al. 2024. Safe Utilization Effect of Passivator, Foliar Inhibitor, and Their

Combined Application on Cadmium-contaminated Farmland. Environmental Science 45 (12): 7237-7244.

Xiao, M., Fan, J., Wang, H., et al. 2022. Effect of Chinese milk vetch combined with lime on cadmium uptake and translocation in rice. China Environmental Science 42, 1: 276-284.

Xiao, R., Huang, Z., Li, X., et al. 2017. Lime and Phosphate Amendment Can Significantly Reduce Uptake of Cd and Pb by Field-Grown Rice. Sustainability 9 (3): 430; https://doi.org/10.3390/su9030430

Xie, Y., Ji, X., Tian, F., et al. 2017. Effect of passivator on Cd uptaking of rice in different Cd pollution characteristics paddy soils. Chinese Journal of Environmental Engineering 11 (02).

Yan, D., Guo, Z., Huang, F., et al. 2020. Effect of Calcium Magnesium Phosphate on Remediation Paddy Soil Contaminated with Cadmium Using Lime and Sepiolite. Environmental Science 8, 41 (3): 1491-1497. https://doi.org/10.13227/j.hjkx.201909095

Yang, D., Li, X., Zhou, Y., et al. 2021. Effects of straw returning with different lime dosages on Cd accumulation in rice. Journal of Agro-Environment Science 40, 06. http://dx.doi.org/10.11654/jaes.2020-1137LI

Yang, R., Chen, X., Zhang, Y., et al. 2021. Effects of Nano Material on Cadmium Accumulation Capacity and Grain Yield of Indica Hybrid Rice Under Wetting-drying Alternation Irrigation. Environmental Science 42, 01. https://doi.org/10.13227/j.hjkx.202005182

Yuan, C., Li, B., Zhu, R., et al. 2019. Immobilization of Cd and Pb using different amendments of cultivated soils around lead-zinc mines. Journal of Agro-Environment Science 38 (04).

Yuan, F., Tang, X., Wu, J., et al. 2021. Simultaneous immobilization of arsenic and cadmium in paddy soil by Fe-Mn binary oxide: A field-scale study. Environmental Science 42, 7: 3535-3548. https://doi.org/10.13227/j.hjkx.202010183

Yang, Y., Chen, J., Huang, Q., et al. 2018. Can liming reduce cadmium (Cd) accumulation in rice (Oryza sativa) in slightly acidic soils? A contradictory dynamic equilibrium between Cd uptake capacity of

roots and Cd immobilisation in soils. Chemosphere 193: 547-556. https://doi.org/10.1016/j.chemosphere.2017.11.061

Zeng, T., Khaliq, M.A., Li, H., et al. 2020. Assessment of Cd availability in rice cultivation (Oryza sativa): Effects of amendments and the spatiotemporal chemical changes in the rhizosphere and bulk soil. Ecotoxicology and Environmental Safety 196: 110490. https://doi.org/10.1016/j.ecoenv.2020.110490

Zhang, J., Kong, F., Lu, S. 2022. Remediation Effect and Mechanism of Inorganic Passivators on Cadmium Contaminated Acidic Paddy Soil. Environmental Science 43(10): 4679-4686. https://doi.org/10.13227/j.hjkx.202112273

Zhang, S., Quan, L., Zhu, Y., et al. 2020. Differential effects of three amendments on the immobilisation of cadmium and lead for Triticum aestivum grown on polluted soil. Environmental Science and Pollution Research 27: 40434-40442. https://doi.org/10.1007/s11356-020-10079-6

Zhang, Q., Zhang, L., Liu, T., et al. 2018. The influence of liming on cadmium accumulation in rice grains via iron-reducing bacteria. Science of The Total Environment 645: 109-118. https://doi.org/10.1016/j.scitotenv.2018.06.316

Zhang, Z., Ji, X., Xie, Y., et al. Effects of quicklime application at different rice growing stage on the cadmium contents in rice grain. Journal of Agro-Environment Science 35 (05).

Zhao, Y., Zhang, C., Wang, C., et al. 2020. Increasing phosphate inhibits cadmium uptake in plants and promotes synthesis of amino acids in grains of rice. Environmental Pollution 257: 113496. https://doi.org/10.1016/j.envpol.2019.113496

Zhou, L., Xiao, F., Xiao, H., et al. 2021. Effects of Lime on Cadmium Accumulation of Double-Season Rice in Paddy Fields with Different Cadmium Pollution Degrees. Scientia Agricultura Sinica 54 (4): 780-791. https://doi.org/10.3864/j.issn.0578-1752.2021.04.010

Zhu, Q., Huang, D., Zhu, G., et al. 2008. Sepiolite is recommended for the remediation of Cd-contaminated paddy soil. Acta Agriculturae Scandinavica, Section B-Soil & Plant Science 60 (2): 110–116.

https://doi.org/10.1080/09064710802672624
