# Supplementary figures and images for: Mechanistic Pathways Controlling Cadmium Bioavailability and Ecotoxicity in Agricultural Systems: A Global Meta-Analysis of Lime Amendment Strategies
Source: Biology (Basel). 2026 Jan 23;15(3):207. doi: 10.3390/biology15030207 (PMC12896412; doi:10.3390/biology15030207)

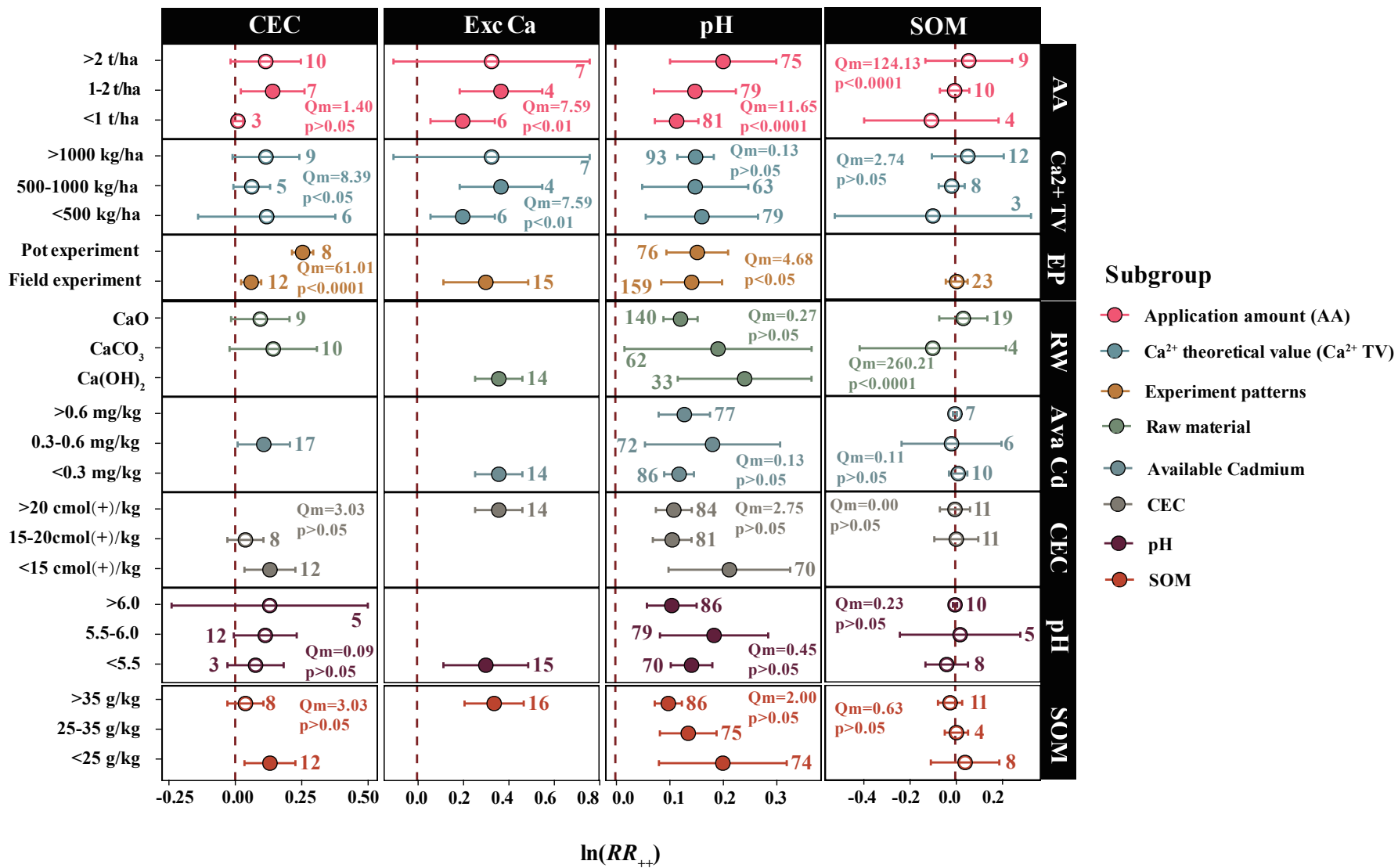

Supplement: Supplementary file 1 [file biology-15-00207-s001.zip › Figure.S3.pdf]
